# Rectification ratio based determination of disulfide bonds of β2 extracellular loop of BK channel

**DOI:** 10.1080/19336950.2018.1551660

**Published:** 2018-11-26

**Authors:** Xiying Guo, Haowen Liu, Zhigang Huang, Yanting Wang, Yan Zhang, Lu-Yang Wang, Chunyang Cao, Sheng Wang, Jiuping Ding

**Affiliations:** a Key Laboratory of Molecular Biophysics of the Ministry of Education, College of Life Science and Technology, Huazhong University of Science and Technology, Wuhan, China; b State Key Laboratory of Bio-organic and Natural Product Chemistry, Shanghai Institute of Organic Chemistry, Chinese Academy of Sciences, Shanghai, China; c Program in Neurosciences and Mental Health, SickKids Research Institute and Department of Physiology, University of Toronto, Toronto, Canada

**Keywords:** BK channel, disulfide bond, rectification ratio, β2 subunit

## Abstract

Large-conductance Ca^2+^-activated K^+^ (BK) channels are composed of a pore-forming α and a variable number of auxiliary β subunits and play important roles in regulating excitability, action potential waveforms and firing patterns, particularly in neurons and endocrine and cardiovascular cells. The β2 subunits increase the diversity of gating and pharmacological properties. Its extracellular loop contains eight cysteine residues, which can pair to form a high-order structure, underlying the stability of the extracellular loop of β2 subunits and the functional effects on BK channels. However, how these cysteines form disulfide bonds still remains unclear. To address this, based on the fact that the rectification and association of BK α to β2 subunits are highly sensitive to disruption of the disulfide bonds in the extracellular loop of β2, we developed a rectification ratio based assay by combining the site-directed mutagenesis, electrophysiology and enzymatic cleavage. Three disulfide bonds: C1(C84)-C5(C113), C3(C101)-C7(C148) and C6(C142)-C8C(174) are successfully deduced in β2 subunit in complex with a BK α subunit, which are helpful to predict structural model of β2 subunits through computational simulation and to understand the interface between the extracellular domain of the β subunits and the pore-forming α subunit.

## Introduction

Large-conductance Ca^2+^-activated K^+^ (BK) channels, consisting of a tetramer of *Slo1* α subunits, are ubiquitously expressed in many tissues. They play critical roles in modulating physiological activities, such as neurotransmitter release and endocrine secretion from neurons or endocrine cells, contraction in smooth muscle cells and even frequency tuning in hair cells [1–7]. Native BK-type channels are often associated with tissue-specific auxiliary β1−β4 subunits. These auxiliary subunits share a similar topology of two transmembrane (TM1 and TM2) segments, intracellular N and C terminals, and a large extracellular loop [8–12]. The β subunits apparently increase the Ca^2+^ sensitivity of *Slo1* α channels, modify the channel kinetics and alter their pharmacological properties [6,8,9,13–19]. Among them, the β2 subunit was primarily found in rat chromaffin cells, pancreatic β cells and DRG neurons [17,20]. It can facilitate channel activation, but it has no effects on the voltage sensitivity of the channel [21]. Its N-terminus is hydrophobic, which induces a rapid N-type inactivation of BK currents and increases the calcium sensitivity of BK channels [19,22–24]. However, it prevents the scorpion toxin charybdotoxin (ChTX) from approaching the channel pore [17]. It produces nonlinearity in the instantaneous current-voltage (I-V) curve of the resulting BK currents, which was termed as rectification [25,26]. The nonlinearity was attributed to the lysine-rich rings in the extracellular loop of β2.

The extracellular loop of β2, β3, β4 subunits is highly sophisticated with eight cysteines and multiple N-glycosylation sites; thus, the loop structure is too hard to be resolved by NMR or X-ray techniques. It is even more difficult to resolve the correctly folded β2 subunit independently because the subunit cannot reach the membrane surface alone without an associated α subunit [27,28]. The eight cysteines of the β2 extracellular loop were suggested to form four disulfide bonds [29], which intrinsically fix the global folding of the β2 extracellular loop, thus determining its biological functions. However, how these cysteines in the extracellular loop of β2 subunit form disulfide bonds still remains unclear. To address this, in this report, after we failed to get the soluble extracellular loop of β2 subunit through overexpression in *E. coli* system, and could not directly determine disulfide bonds with mass spectrum assay, we developed a novel technique based on the changes of the rectification ratio (*R*). This assay composes two steps, the first one is to perform single-site and double-site mutations from residue Cys to Ser in the extracellular loop of β2, minimally reducing the changes in the channel conformation and kinetics of BK (β2) currents; the second one is to use TEV protease digestion to verify the above disulfide pairing mode. Three disulfide bonds cysteine pairs are formed successfully deduced: C1(C84)-C5(C113), C3(C101)-C7(C148) and C6(C142)-C8(C174). To elucidate the unique properties imparted by the β2 subunit, these structural information were further docked into the three-dimensional (3D) structure mSlo1 in complex with β2, which should be helpful in disgning new drug and verifying the crystal structure of β2 in the future.

## Results

### Basic properties of BK(mSlo1+β2) channels induced by β2 subunits

**Figure 1.**
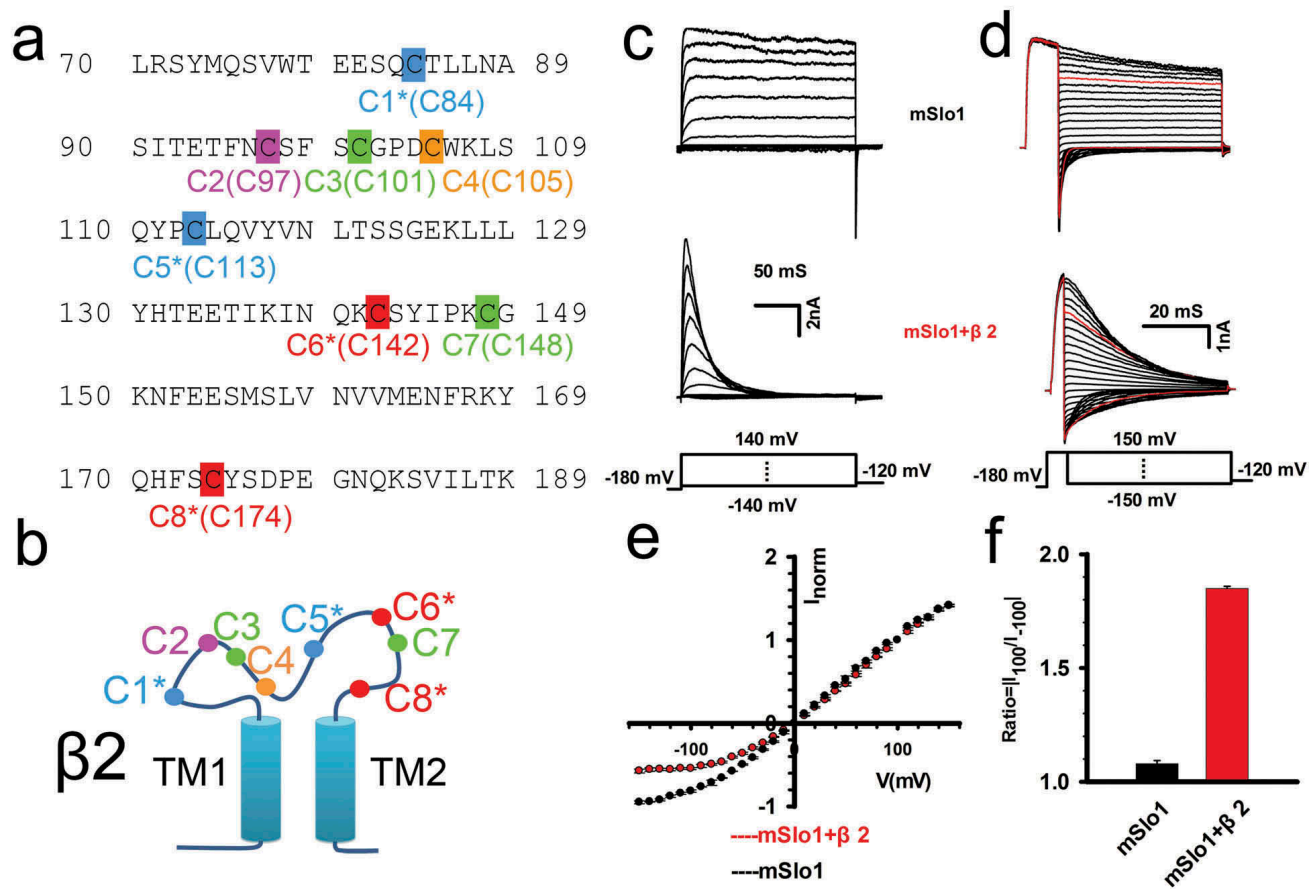
Rectification characteristics of mSlo1 and mSlo1+ β2 channels. (a)The amino acid sequence of the extracellular β2 loop，in which cysteine were highlighted, the conserved cysteines were labeled with an asterisk *. (b) Schematic representation of β2. The β2 subunit is composed of a large extracellular loop, two transmembrane domains (TM1, TM2), and N- (inactivation domain) and C-termini. (c) Representative traces of mSlo1 (top panel) and mSlo1+ β2 (middle panel) channels recorded from inside-out patches from HEK293 cells. Currents were elicited at potentials from −140 mV through 140 mV in increments of 20 mV, after a prepulse to remove inactivation, in the presence of 160 mM *K*_o_^+^/160 mM *K*_i_^+^ with 10 μM Ca^2+^. The voltage protocol is plotted in the bottom panel. Scale bars represent 50 ms and 2 nA, as indicated. (d) Traces were obtained from the same patch as shown in the left panel in C. Instantaneous tail currents of mSlo1 and mSlo1+ β2 were activated by steps of voltages ranging from −150 to 150 mV with an increment of 10 mV after a 4 or 10 ms prepulse of 150 mV in the presence of 10 μM Ca^2+^. The voltage protocol is plotted at the bottom. The red lines represent the currents activated at either +100 mV or −100 mV. Scale bars represent 20 ms and 2 nA, as indicated. (e) The instantaneous tail currents of mSlo1 (black circle) and mSlo1+ β2 (red circle), after normalization to the tail current at +100 mV, are plotted as a function of voltage. (f) The histogram of the rectification ratios R = |I_100_/I_−100_| is plotted for mSlo1 and mSlo1+ β2. The ratios are 1.12 ± 0.01 (n = 10) and 1.85 ± 0.02 (n = 12) for mSlo1 and mSlo1+ β2, respectively. Statistical significance for the data was determined using two-tailed unpaired Student’s t-test (**P < 0.01).

To establish the potential contributions of cysteine pairings to the structural conformation of the β2 extracellular loop, we examined the electrophysiological characteristics of BK(β2) channels (Figure 1(a-b)). Upon binding with the auxiliary β2 subunits, the mSlo1 α subunit forms functional BK channels that typically exhibit a rapid and complete inactivation with strong instantaneous outward rectification [16,17,30,31]. As shown in Figure 1(c), representative mSlo1 and mSlo1+ β2 currents were recorded from an inside-out patch at 10 μM of Ca^2+^ ion. In contrast to the non-inactivating mSlo1 currents, the currents recorded upon β2 being incorporated showed significant acceleration of the inactivation time course under symmetrical 160 mM of K^+^ solutions (e.g., time constant τ_i_~20 ms at 100 mV; half-maximal activation voltage of mSlo1+ β2 channel (V_50_)~-15 mV). Under such conditions, we further recorded and compared the tail currents of the mSlo1 and the mSlo1+ β2 activated by a 10 ms (for mSlo1) or 4 ms (for mSlo1+ β2) voltage step to 150 mV from a holding potential of −180 mV, followed by steps to potentials from – 150 to 150 mV, in the presence of 10 μM of Ca^2+^ (Figure 1(d)). The normalized instantaneous tail currents were plotted as a function of repolarization potential for mSlo1 and for mSlo1+ β2 (Figure 1(e)). Consistent with the results from previous work [24,26], the instantaneous current-voltage (I-V) curve of the mSlo1+ β2 channel showed nonlinearity, which markedly differed from the mSlo1 channel alone. The instantaneous I-V curves normalized to I (+100 mV) were used to determine the rectification property of mSlo1+ β2 channels.

To quantitatively describe the nonlinearity behavior, we introduced a term i.e. rectification ratio R = |I_100_/I_−100_|. The red traces (±100 mV) were used to calculate the rectification ratios as shown in Figure 1(d). The statistics in Figure 1(f) displayed that the *R*_α_ was equal to 1.12 ± 0.01 and *R*_β2_ was determined as 1.85 ± 0.02, which are almost identical to the reported results [26]. As described previously, the instantaneous outward rectification of mSlo1+ β2 should correspond to intrinsic structural characteristics of BK(mSlo1+ β2) channels, reflecting a specific charge-distribution pattern of the extracellular loop of β2, *i.e*., lysine residues that are thought to electrostatically impede K^+^ permeation [26].

### *The disulfide bonds affect the rectification ratio* R

**Figure 2.**
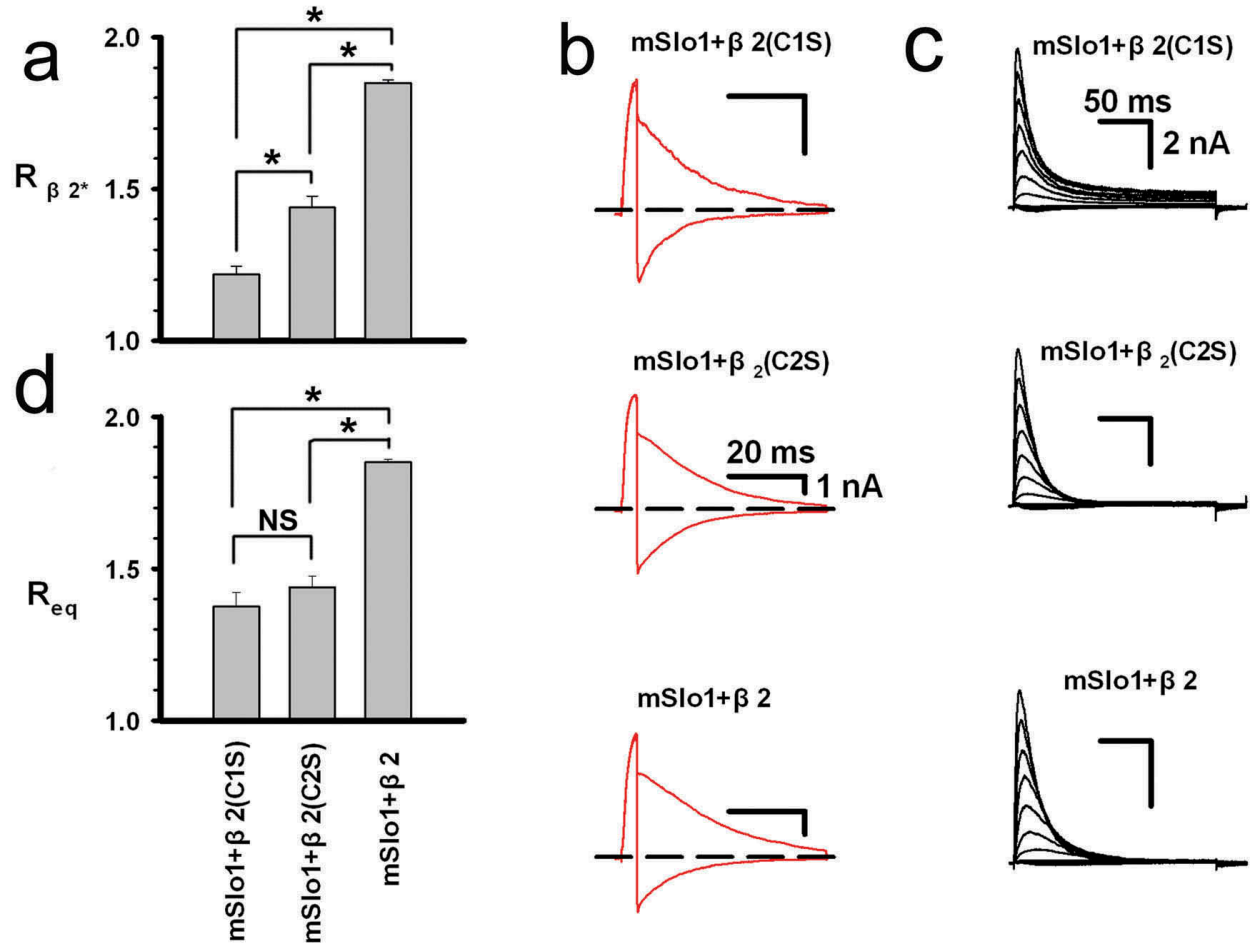
The rectification ratio R was modified to Req to remove the unbinding effect of α and β. (a) The experimental rectification ratios *R*_β2*_ are plotted for mSlo1+ β2(C1S), mSlo1+ β2(C2S), and mSlo1+ β2, as indicated on the bottom of Figure 4(d). The ratios are 1.22 ± 0.03 (n = 8), 1.43 ± 0.04 (n = 7), 1.85 ± 0.05 (n = 12) for mSlo1+ β2(C1S), mSlo1+ β2(C2S), and mSlo1+ β2, respectively. (b) The instantaneous tail currents of mSlo1+ β2(C1S), mSlo1+ β2(C2S), and mSlo1+ β2 were activated by steps in voltages ranging from −150 to 150 mV with an increment of 10 mV after a 4 ms prepulse of 150 mV in the presence of 10 μM Ca^2+^. The voltage protocol is the same as shown in Figure 1(d), and the scale bars represent 20 ms and 1 nA. The figure only shows the currents activated at +100 mV and −100 mV. (c) Currents were elicited at potentials from −140 mV to 140 mV in an increment of 20 mV, after a prepulse to remove inactivation, in the presence of 160 mMK_o_^+^/160 mM K_i_^+^ with 10 μM Ca^2+^. The voltage protocol and scale bars are the same as shown in Figure 1(c). (d) The equivalent rectification ratios *R*_eq_ are plotted for mSlo1+ β2(C1S) (*i.e., R*_eq_(C1S)), mSlo1+ β2(C2S) (*i.e., R*_eq_(C2S)) and mSlo1+ β2, as indicated. These ratios are 1.38 ± 0.03 (n = 8), 1.43 ± 0.04 (n = 7) and 1.85 ± 0.05 (n = 12) for mSlo1+ β2(C1S), mSlo1+ β2(C2S) and mSlo1+ β2, respectively. Statistical significance for all data was determined using one-way ANOVA (*P < 0.05, NS means not significant).

**Figure 3.**
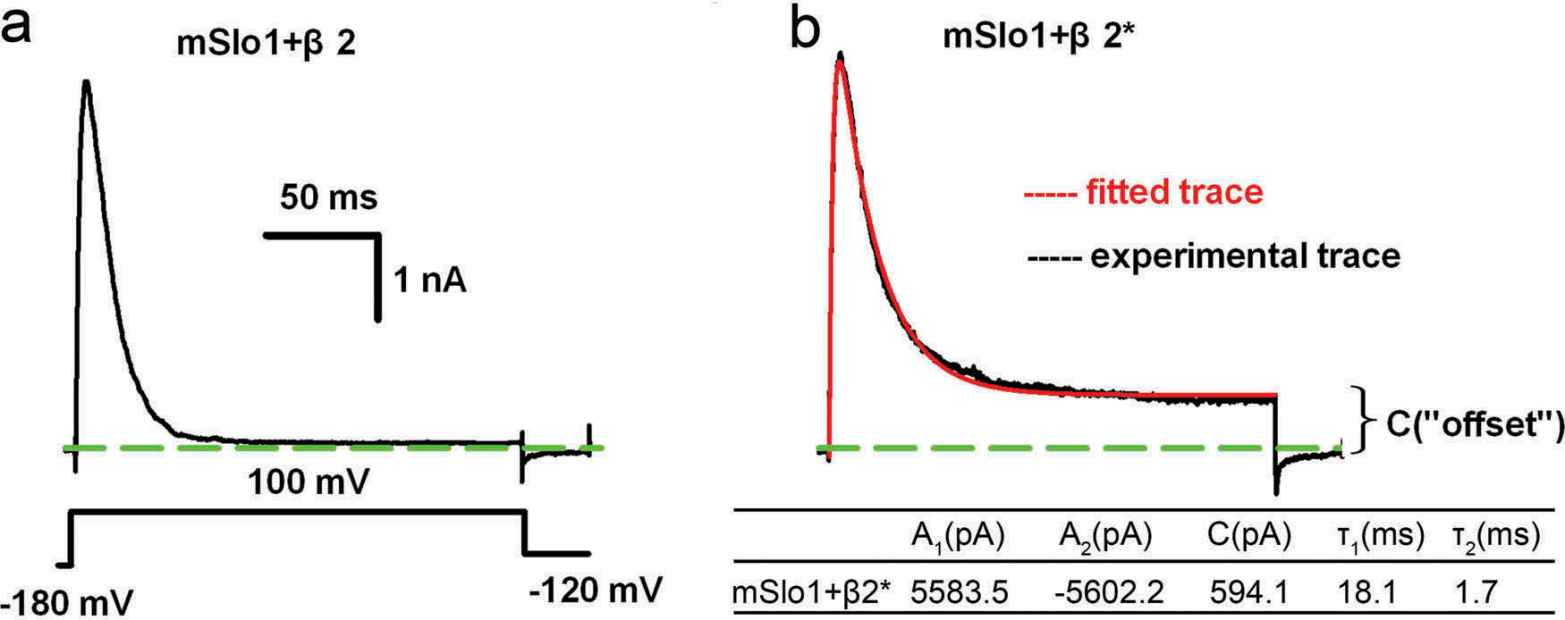
Determination of “offset” of mSlo1+ β2 mutation currents. (a) Representative trace of mSlo1+ β2 channels recorded from inside-out patches from HEK293 cells with the voltage protocol plotted at the bottom. (b) The experimental trace of mSlo1+ β2 mutants (black) recorded from inside-out patches from HEK293 cells and the fitted trace of mSlo1+ β2 mutants are shown in red. The fitting parameters are plotted in a table at the bottom. (a-b) Scale bars represent 50 ms and 1 nA, as indicated.

The Cys-Cys (C-C) pairing mode is important to the folding of the β2 loop; thus the charge distribution pattern of the extracellular loop of β2 heavily depends on the disulfide bonds. Moreover, the lysine residues in the β2 loop were suggested to be tethered in close spatial proximity to form three stable rings with electric charges above the channel pore [26](Figure 1(a-b)), the perturbation of the C-C pairings can thus destabilize the conformation of the charged ring and further affects the rectification characteristics of BK(β2) currents (*i.e., R*_β2_ = |I_100_/I_−100_|). To explore the effect of the C-C pairing mode on the β2 conformation, we changed cysteine into serine (C→S). The values of *R*_β2*_^C1S^ and *R*_β2*_^C2S^ of the mutants were observed to be obviously less than that of the wild-type *R*_β2_, as we expected (Figure 2(a-b)). The C2S mutant had been completely inactivated, while the C1S mutant displayed incomplete inactivation (Figure 2(b-c)), indicating that the association of α subunit to β2 subunit was reduced due to the mutation from cysteine to serine. The decrease in association further resulted in a larger variance of *R*_β2*_ value. This observation was consistent with the changes in the degree of incomplete inactivation (“offset”), as shown in Figure 3, which heavily depended on its steady-state component. As described in Methods and Materials, the modified algorithm for the incomplete inactivation was then developed to calculate the association of α subunit to β2 subunit, and the equivalent rectification ratios, *R*_eq_, were further introduced to minimize the variance. The modified rectification ratio *R*_eq_ was shown in Figure 2(d), where *R*_eq_ was significantly lower in both mutants than that in wild-type protein.

**Figure 4.**
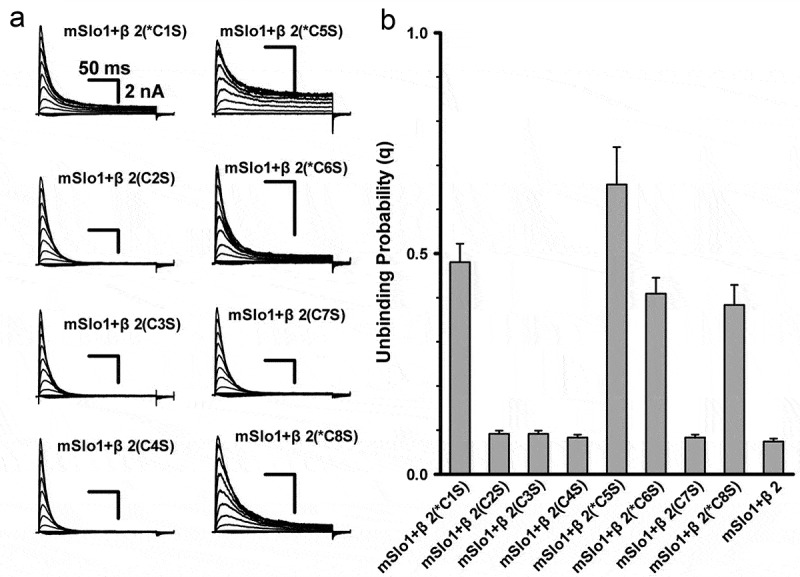
The mutations of conserved cysteines caused the association of α and β reduced. (a)The representative traces are for mSlo1+ β2(C1S), mSlo1+ β2(C2S), mSlo1+ β2(C3S), mSlo1+ β2(C4S), mSlo1+ β2(C5S) and mSlo1+ β2(C6S), mSlo1+ β2(C7S) and mSlo1+ β2(C8S), as indicated. The voltage protocol and scale bars are the same as shown in Figure 1(c). (b) The unbinding probability (q) is plotted for mSlo1+ β2 (C1S), mSlo1+ β2 (C2S), mSlo1+ β2 (C3S), mSlo1+ β2 (C4S), mSlo1+ β2 (C5S), mSlo1+ β2 (C6S), mSlo1+ β2 (C7S) and mSlo1+ β2 (C8S) and mSlo1+ β2, as indicated. The probabilities are 0.48 ± 0.04 (n = 5) for mSlo1+ β2 (C1S), 0.09 ± 7.4e-3 (n = 6) for mSlo1+ β2 (C2S), 0.09 ± 7.5e-3 (n = 7) for mSlo1+ β2 (C3S), 0.08 ± 6.6e-3 (n = 7) for mSlo1+ β2 (C4S), 0.66 ± 0.08 (n = 4) for mSlo1+ β2 (C5S), 0.41 ± 0.04 (n = 4) for mSlo1+ β2 (C6S), 0.08 ± 6.6e-3 (n = 8) for mSlo1+ β2 (C7S), 0.38 ± 0.05 (n = 4) for mSlo1+ β2 (C8S) and 0.07 ± 6.8e-3 (n = 12) for mSlo1+ β2. Statistical significance for all data was determined using one-way ANOVA. The comparison of the four conserved cysteine mutations (C1S, C5S, C6S, and C8S) vs. β2 (WT) showed significant differences (P < 0.05), whereas the comparison of the other four cysteine mutations (C2S, C3S, C4S, and C7S) vs. β2 (WT) did not. The results of the comparisons between β2 and C1S,C5S, C6S, and C8S were not significant (P > 0.05).

To identify whether or not the two cysteines, e.g., C_A_ and C_B_, form a disulfide bond, the changes in the values of rectification ratios resulted from single-site and double-site mutations will be examined. If a disulfide bond is formed, the *R*_eq_ values of the three mutants (*i.e*., C_A_S, C_B_S and C_A_S/C_B_S) will show almost similar changes because these mutations interrupt the same disulfide bond. Otherwise, the changes in *R*_eq_ values of a single-site mutant are different from those of the double-site mutant (*i.e*., C_A_→S/C_B_→S), indicating that C_A_ and C_B_ do not form a disulfide bond.

### Two subgroups of cysteine mutants

The currents from all eight single-site directed cysteine mutants were recorded, indicating that the mutations led to inactivation in very different extents (Figure 4). On the basis of the extent of inactivation, the mutations were divided into two groups, termed as conserved and non-conserved mutations, respectively, so that we could narrow down the candidates of potentially coupled cysteine pairs with similar channel-gating characteristics. Accordingly, the pairing mode was firstly determined based on the changes in the rectification ratios (*R*_eq_) produced by the electrostatic fields from the charged amino acids surrounding the cysteines or directly by the C→S mutations. Then, to support our hypothesis, we performed conventional protease digestion (Figure S1).

As shown Figure 4(a), the mutations on four conserved cysteines (the corresponding mutants were labeled with the star *, *i.e*., residues *C1, *C5, *C6 and *C8) only resulted in incomplete inactivation. To confirm whether or not these mutants could target membrane, we co-expressed them with mSlo1 respectively, and extracted the membrane fractions for western blot. We found that mutants C1S, C5S, C6S and C8S could be localized to the membrane, albeit lower membrane expression level than pWT β2 (*i.e*. β2-HA) (Figure S2). These results are consistent with the results from electrophysiological experiments. Our previous studies [30] showed that BK(β2) channels displayed incomplete inactivation, indicating a decrease in the binding probability p of mSlo1 and β2. The probability could be calculated using the unbinding probability q (= 1–p). The q values calculated for each mutant were divided into the conserved and non-conserved groups, the higher q group (C1S, C5S, C6S and C8S) and the lower q group (C2S, C3S, C4S and C7S), as shown in Figure 4(b). Furthermore, we analyzed the values of V_50_, the inactivation time constants of BK(β2) and all the mutations (Table S1). A similar pattern was observed: the values of V_50_ of the mutants C1S, C5S, C6S and C8S were in the range of positive values, while V_50_ of other mutants were close to that of the wild type protein, *i.e*. hovering around −12.7 mV. At the same time, the inactivation time constants of the mutants C1S, C5S, C6S and C8S were about 25 ms, while those of other mutants were close to that of that of the wild type protein, *i.e*. values of these two parameters of all mutants varied from each other, the extent of variation was very narrow. These results also indicated that these cysteine mutations disturbed the conformation of β2 in a slight degree, and the mutants could still hold the basic electrophysiological properties of BK(β2) channels. All these results demonstrated that the potential pairs might be formed within each subgroup.

### *Rectification ratio* R *based determining of disulfide-bonds*

**Figure 5.**
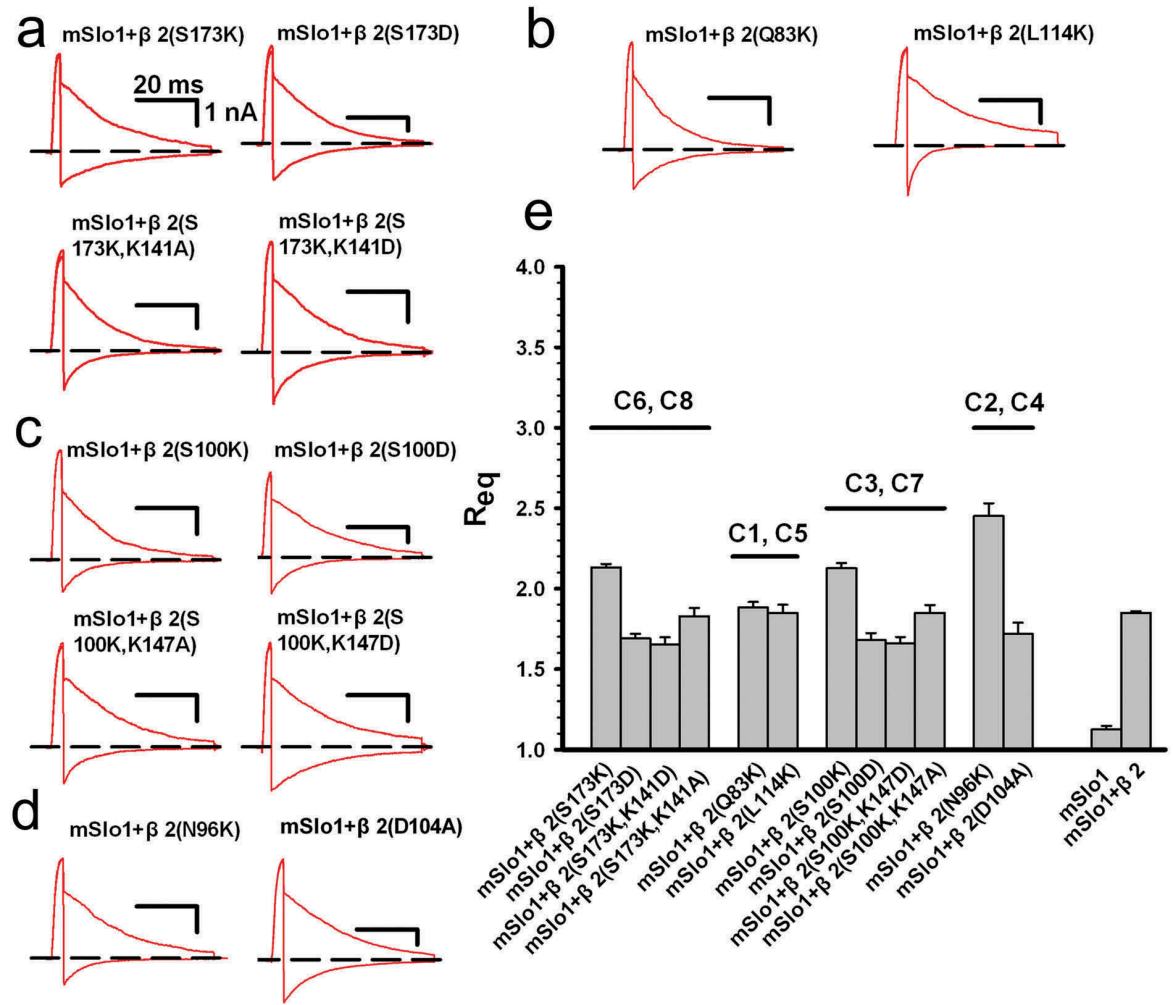
The Req characteristics induced by the charged residues surrounding cysteines. (a)The representative instantaneous tail traces were plotted for mSlo1+ β2 (S173K), mSlo1+ β2 (S173D), mSlo1+ β2 (S173K/K141A) and mSlo1+ β2 (S173K/K141D), as indicated. (b) The representative instantaneous tail traces are plotted for mSlo1+ β2 (Q83K) and mSlo1+ β2 (L114K), as indicated. (c) The representative instantaneous tail traces are plotted for mSlo1+ β2 (S100K), mSlo1+ β2 (S100D), mSlo1+ β2 (S100K/K147D) and mSlo1+ β2 (S100K/K147A), as indicated. (d) The representative instantaneous tail traces are plotted for mSlo1+ β2 (N96K) and mSlo1+ β2 (D104A), as indicated. (A), (B), (C) and (D) were stimulated using the same voltage protocol, and the scale bars are the same as shown in Figure 2(d). (e) The equivalent rectification ratios *R*_eq_ are plotted for mSlo1+ β2 (S173K), mSlo1+ β2 (S173D), mSlo1+ β2 (S173K/K141A), mSlo1+ β2 (S173K/K141D), mSlo1+ β2 (Q83K), mSlo1+ β2 (L114K), mSlo1+ β2 (S100K), mSlo1+ β2 (S100D), mSlo1+ β2 (S100K/K147D), mSlo1+ β2 (S100K/K147A), mSlo1+ β2 (N96K), mSlo1+ β2 (D104A), mSlo1 and mSlo1+ β2, as indicated. These ratios are 2.13 ± 0.02 (n = 8) for mSlo1+ β2 (S173K), 1.69 ± 0.02 (n = 6) for mSlo1+ β2 (S173D), 1.83 ± 0.05 (n = 8) for mSlo1+ β2 (S173K/K141A), 1.65 ± 0.05 (n = 7) for mSlo1+ β2 (S173K/K141D), 1.88 ± 0.03 (n = 8) for mSlo1+ β2 (Q83K), 1.85 ± 0.05 (n = 7) for mSlo1+ β2 (L114K), 2.13 ± 0.03 (n = 8) for mSlo1+ β2 (S100K), 1.68 ± 0.04 (n = 7) for mSlo1+ β2 (S100D), 1.69 ± 0.04 (n = 6) for mSlo1+ β2 (S100K/K147D), 1.85 ± 0.05 (n = 6) for mSlo1+ β2 (S100K/K147A), 2.45 ± 0.08 (n = 6) for mSlo1+ β2 (N96K), 1.71 ± 0.07 (n = 8) for mSlo1+ β2 (D104A), 1.15 ± 0.04 (n = 10) for mSlo1, and 1.85 ± 0.05 (n = 12) for mSlo1+ β2. Statistical significance for all data was determined using one-way ANOVA, and the results were significant (p < 0.05), except for the mSlo1+ β2 (S173D) vs. mSlo1+ β2 (S173K/K141D), mSlo1+ β2 (S173K/K141A) vs. mSlo1+ β2, mSlo1+ β2 (Q83K) vs. mSlo1+ β2 (L114K), mSlo1+ β2 (S100D) vs. mSlo1+ β2 (S100K/K147D), and mSlo1+ β2 (S100K/K147A) vs. mSlo1+ β2 comparisons.

To confirm that the potential disulfide bonds formed in subgroup, we performed the mutations on the residues around the cysteine, as shown in Figure 5. As we know, the rectification ratio originates from three rings containing positively charged residues (lysine) around the pore; the electrostatic field of these rings decreases the local K^+^ concentration to cause rectification [26]. Assuming the pairing mode of C_A_-C_B_, the change in the charge number (Δq_e_) on either the C_A_ or the C_B_ side should cause a similar change in *R*_eq_. The larger the Δq_e_, the larger the *R*_eq_. In the case of C6 and C8, for example, there is a total positive charge number of q_e_ = 1 (per subunit) in the region near to C6 (*i.e*., K_141_C_142_S_143_) and C8 (*i.e*., S_173_C_174_Y_175_). Compared with the value of *R*_eq_^β2^ (equal to 1.85) of wild type BK(mSlo1+ β2), the *R*_eq_^S173K^ value was increased to 2.13 due to Δq_e_ = 1, the *R*_eq_^S173D^ value was decreased to 1.69 due to Δq_e_ = −1, the *R*_eq_ value of β2^S173K,/K141A^ returned to 1.83 due to Δq_e_ = 0, and the *R*_eq_^S173K^^/K141^^D^ value was decreased to 1.65 due to Δq_e_ = −1. These results suggested that C6 and C8 were located near the channel pore, and had the best opportunity to form a disulfide bond. Similarly, in the case of C1 and C5 (Figure 1(a)), there are no charged residues (q_e_ = 0) near C1 and C5. With Δq_e_ = 1 per subunit, the two mutants β2(Q83K) and β2(L114K) yielded the values of *R*_eq_^Q83K^ equal to 1.88 and *R*_eq_^L114K^equal to 1.85, respectively; which were close to that of WT β2. This observation suggested that residues C1 and C5 might pair together; these residues are far from the pore region and strongly impact the rectification characteristics of BK(β2) channels.

To determine how the remaining four cysteine residues C2, C3, C4 and C7 form disulfide bonds(Figure 1(a)), we mutated the residues N96, S100, D104 and K147 close to them and separated them into two groups, C3-C7 and C2-C4, as shown in the right panel of Figure 5. For group C3-C7, *R*_eq_^S100K/K147A^ ≈ *R*_eq_^β2^ (where Δq_e_ = 0); *R*_eq_^S100K^> *R*_eq_^β2^ (where Δq_e_ = +1), and *R*_eq_^S100K/K147D^ = *R*_eq_^S100D^ < *R*_eq_^β2^ (where Δq_e_ = – 1). Therefore, residues C101 and C148 were paired together. For group C97-C105, *R*_eq_^D104A^ (where Δq_e_ = +1) was almost identical to *R*_eq_^β2^ and was much smaller than *R*_eq_^N96K^ (where Δq_e_ = +1), indicating that residue D104 of β2 is not near the pore because it is insensitive to the charge variation. Additionally, given that N96 is a typical N-linked glycosylation site [32], we deduced that N96 might cause an anomaly in the channel rectification, and also impede the pairing of C2 and C4.

**Figure 6.**
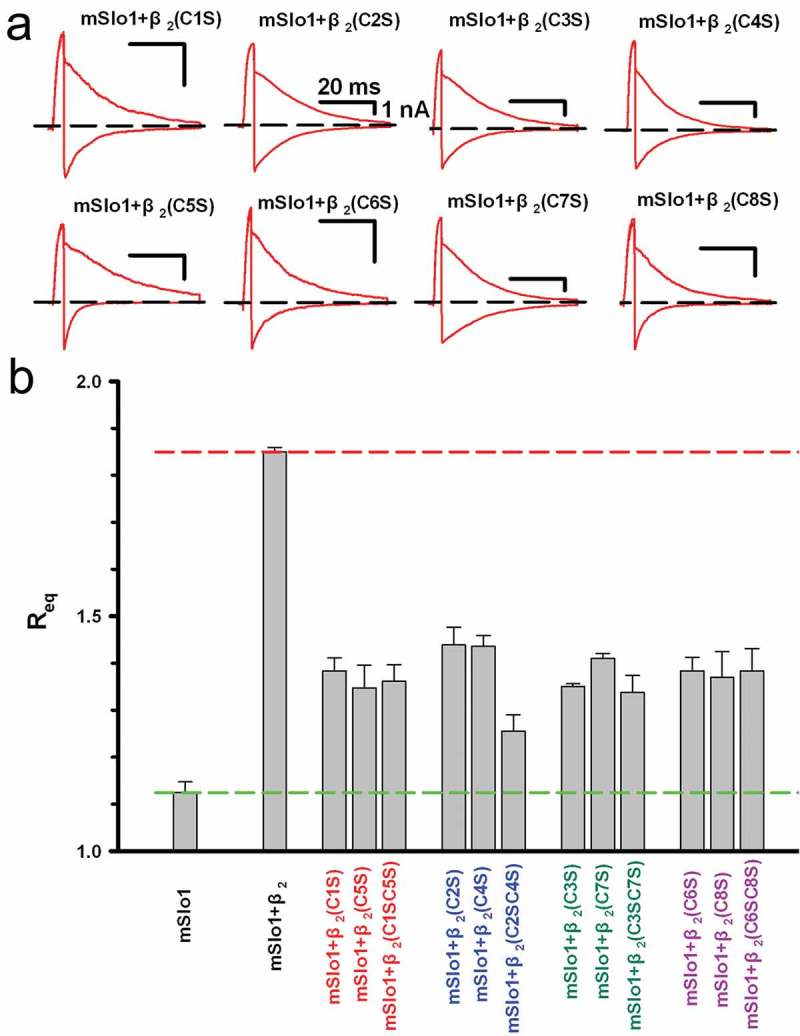
The representative instantaneous tail traces and Req values of the C→S mutations of the above putative Cys-Cys pairs. (a) The representative instantaneous tail traces are plotted for mSlo1+ β2(C1S), mSlo1+ β2(C2S), mSlo1+ β2(C3S), mSlo1+ β2(C4S), mSlo1+ β2(C5S) and mSlo1+ β2(C6S), mSlo1+ β2(C7S) and mSlo1+ β2(C8S). The voltage protocol and scale bars are the same as shown in Figure 2(d), and the figure only shows the currents activated at +100 mV and −100 mV. (b) Comparison of the *R*_eq_ values of the C→S mutations of the above putative Cys-Cys pairs. Except for the two single bars of mSlo1 and mSlo1+ β2, as indicated, each group was composed of three bars, as indicated. The statistical significance of each group was determined using one-way ANOVA, and the result was not significant (p > 0.05), except for the β2 (C1S, C4S) group result.

As noted above, the pairing criterion for two cysteines is that the *R*_eq_ values of the mutants should be similar in all cases. In Figure 6(a), all the representative tail-current traces of the eight single site-directed mutations from cysteine to serine were recorded in inside-out patches at ±100 mV in the presence of 10 μM Ca^2+^. All the inward currents were obviously smaller than the outward currents, indicating that the rectification ratios of the mutants were larger than 1 with no significant difference. The *R*_eq_ values shown in Figure 6(b) were only plotted for the single and double site-directed mutations on the cysteines which were putatively paired. Comparing the *R*_eq_ values set by set, we found that the three *R*_eq_ values of all possible pairs, except for that of C2 and C4, were relatively similar within each set, indicating that C2 and C4 were unpaired based on the above criteria. All the *R*_eq_ values combined for the single-sited and double-site directed mutations of the eight cysteines were presented in Figure S3 and Table S2. Taken together, our results suggested three putative cysteine pairs C1(C84)-C5(C113), C3(C101)-C7(C148) and C6(C142)-C8(C174) within the β2 loop (Figure 6(a)).

### Confirmation of the cysteine pairing mode by protease digestion

**Figure 7.**
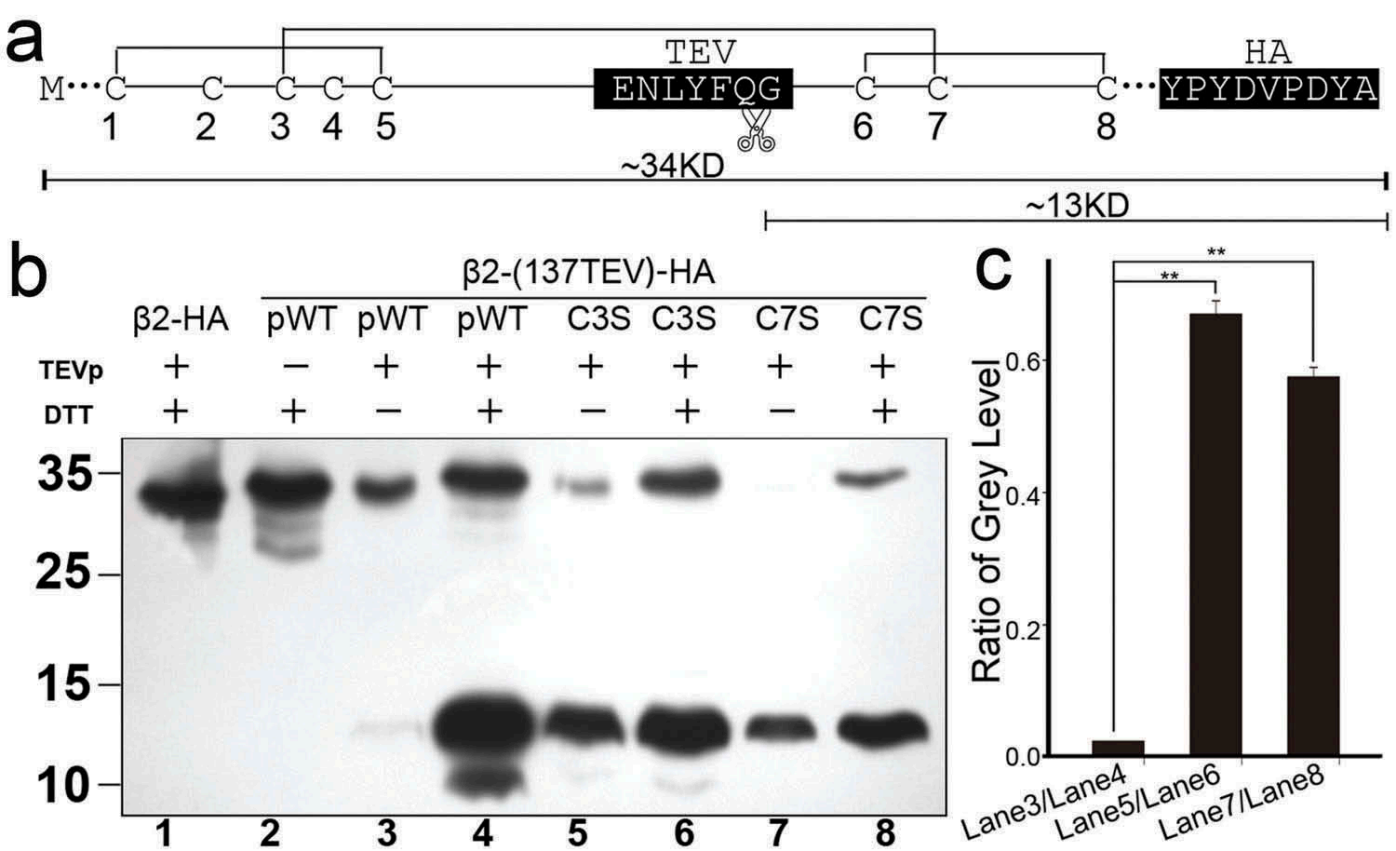
Confirmation of disulfide cross-linking pattern of cysteines in the hβ2 extracellular loop. (a) Predicted cysteine-pairing model of the pWT(β2) tagged with 137TEV and C-HA. The relative positions of the cysteine residues in the hβ2 extracellular loop, the TEV protease recognition site, and the HA tag are labeled as indicated, according to the above electrophysiological experiments. In this model, the molecular weight of pWT β2 is approximately 34 KDa, including the N-linked glycosylation, and the TEV protease proteolytic fragment with the remaining C-terminal HA epitope is approximately 13 KDa. (b) Immunoblot (Ib) with anti-HA antibody showing the fragments containing the C-terminal HA epitope of β2. Top, an approach was designed for TEV enzyme restriction analysis. Left, the predicted molecular weights after digestion of TEV protease with antibody against the C-terminal HA epitope. The same amount of protein was loaded in Lane 3 and Lane 4, Lane 5 and Lane 6, Lane 7 and Lane8. (c) The relative gray levels of the 13 kDa bands between the two lanes of ± DTT are plotted as indicated. Their ratios are 0.024 ± 9.4e-6 (n = 3) for Lane3/Lane4, 0.67 ± 0.020 (n = 3) for Lane5/Lane6 and 0.57 ± 0.015 (n = 3) for Lane 7/Lane 8. Data are shown as the mean ± s.e.m. Statistical significance for the data was determined with Student’s t-test (**P < 0.01).

To validate these pairing modes, we then performed protease digestion. We inserted a TEV protease recognition site (ENLYFQG) after the residue K137, at the same time an epitope HA tag (hemagglutinin, YPYDVPDYA) was attached at the C-terminus (Figure 7(a)). This pWT(β2) construct maintained the basic kinetic characteristics of β2, except for a reduced rectification ratio (Figure S4) caused by an extra acidic residue (glutamic acid) located at the beginning of the TEV sequence. This similarity indicates that the TEV site (next to K137) is also close to the channel pore [26].

With an antibody against the C-terminal HA epitope, the digestion experiment of pWT (β2) was designed to identify the pairing mode (Figure 7(a)). DTT was used as reductive agent to remove disulfide bonds in the protein. pWT (β2) and all of its single-site directed cysteine mutants represented only one band of 34 KDa (Figure S5). During the course of the experiment, in lane 3 of Figure 7(b), only one band with a molecular weight of 34 KDa was shown up for the treatment with TEV protease alone, whereas there was an additional band with a molecular weight of 13 KDa in lane 4 for the treatment with both TEV protease and DTT. These results indicated that one disulfide bond formed between (C1, C2, C3, C4, C5) and (C6, C7, C8), linking the two parts together. Moreover, after digestion, in the presence of (+DTT) or in the absence (-DTT) of DTT, one band with a molecular weight of 13 KDa was always shown up when C3 and C7 were alternatively mutated to serine (lanes 5–8 in Figure 7B). Considering that the digestion experiments for each mutation were performed under exactly same conditions except ± DTT, we normalized the gray-level of the 13 KDa bands of – DTT over that of +DTT (Figure 7(c)); this analysis showed that the gray-level of the 13 kDa bands relative to those of C3S and C7S is significantly different from that of pWT β2. These results indicated that (C1, C2, C3, C4, C5) were not linked with (C6, C7, C8), except for C3-C7.

A series of protease digestion experiments similar to the one shown in Figure 3 were designed for all single-site directed C→S mutants except C3S and C7S (Figure 2).The TEV protease treatment of the mutants C1S, C2S, C4S, C5S, C6S and C8S showed lower gray levels for the 13 KDa bands in the absence of DTT; in contrast, the grey levels of the 13 KDa bands in the presence of DTT were higher (Figure S6). These results were consistent with the results above. C6 and C8 belong to the conserved subgroup (C1, C5, C6 and C8); thus, it is reasonable to conclude that the disulfide bond can only form between C6 and C8 or between C1 and C5. Taking these independent evidences together, we concluded that all the Cys-Cys pairs identified in this study are reasonable and reliable.

Additionally, previous studies using a protease digestion approach showed that the four cysteine residues in the extracellular loop of β1 formed two disulfide bonds [29]. To determine how these cysteines form disulfide bonds, the authors mutated three Glu residues into Gln (*i.e*. E13Q, E50Q, and E143Q). However, in our previous study [33], we found residue E13 in β1 was very important, the mutation of this site caused large changes in the basic properties of BK(β1) channels. Therefore, the assumption that the mutation could not disturb the conformation of β1 seems unconscionable. Here, we conducted similar biochemical experiments on β1 subunits. The inserted TEV recognized site sequence had no effect on the basic properties of BK(β1) channel (*i.e*. current traces and V_50_), so we could speculate that the inserted sequence disturbed the conformation of β1 in only a slight degree. As shown in Figure S7, the conserved cysteines were paired exactly the same as β2, which was clearly different from the published results [29].

### Structural model of the β2 subunits simulated using molecular dynamics (MD)

**Figure 8.**
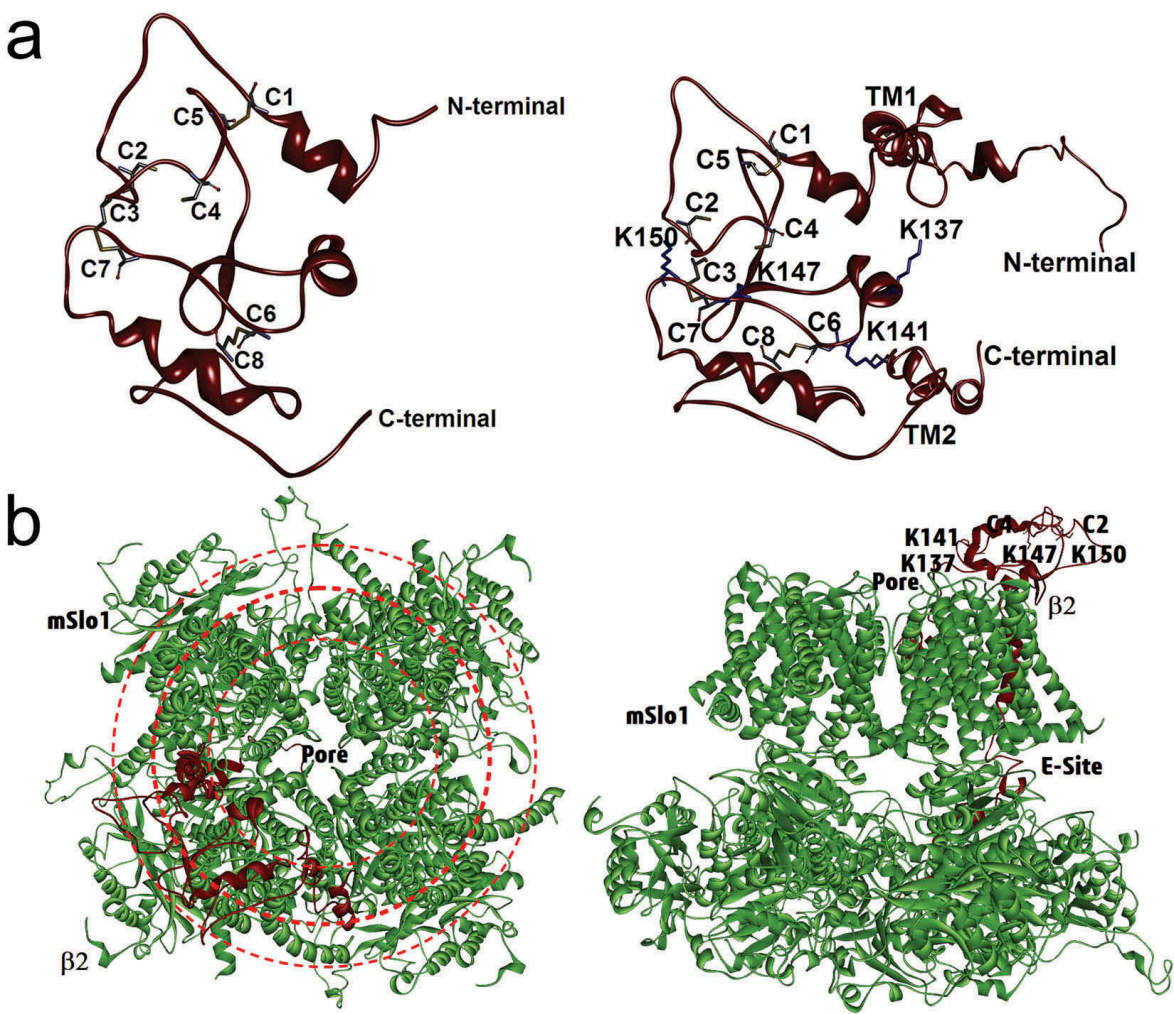
A predicted structure of the β2 subunits simulated using molecular dynamics. For clarity, just one β2 subunit is showed here. (a) Structure of the extracellular loop of β2 found by *de novo* simulation on the basis of the experimental Cys-pairing mode (left) and the detailed structure for the whole assembly of β2 subunit (right). On the right, four basic residues (Lys) are highlighted in stick presentation to show their relative orientation in a β2 subunit. (b) The bird’s eye view (left) and side view (right) of the BK(mSlo1 α/β2) assembly. Left, three rings (red) indicate the external electric field formed by four basic residues lysine located in the extracellular loop of β2. Right, the relative locations of the cysteines and lysines in the β2 loop and an enhancing site (E-site) of mSlo1+ β2 [22].

With the Cys-Cys pairing mode determined, we divided the whole sequence of β2 by cysteines into several fragments that were small enough to be directly predicted by Rosetta suites (version 2.3.1) and studied the conformational features of β2 in the BK channel complex [34]. Using the fact that β2 has a known structure of the N-terminus [35] and a short C-terminus, we could build the whole structure of β2 subunits. With the disulfide bonds constraints, several potential decoys were predicted by MD simulation; these decoys matched the previous experimental results: K137 appeared in a spacious area for the antibody to enter [27] the three lysine rings surrounding the pore [26] and the loci of C2 and C4 near the pore. The final structure of β2 with the highlighted cysteine residues was presented in cartoon form (Figure 8). We noted that the extracellular loop of β2 was composed of three short helices and one short β sheet. K137 was located at the central surface, which left a spacious opening into which an antibody could enter. The structural analysis revealed that most important features of the extracellular loop of β2 satisfied all the above functional conditions (Figure 8 left). Additionally, the structure could explain the difference between C2 and C4 with respect to the orientation of the β2 loop. From the side view of the mSlo1/β2 complex, we surprisingly found that C4 was located quite near C2 (Figure 8 right), possibly forming a pair under certain conditions.

## Discussion

BK channels display diverse properties in many tissues when associated with the auxiliary β subunits (β1-β4), which modulate gating kinetics, including activation, deactivation, inactivation and rectification, as well as pharmacological properties. Given that the extracellular loop of the auxiliary β2 subunit is intimately involved in the unique functional features of BK channels, it is highly desirable to map out its structural information to increase the understanding of how this loop is stabilized to interface with the pore-forming α subunit and generate gating phenotypes.

However, there are usually eight cysteines in the external loop of β subunits, which makes significant difficulty to have a correct protein folding to be detected by either NMR or X-ray possibly due to a wrong pairing mode of cysteines. And there are multiple N-glycosylation sites in the extracellular loop of β subunits, which makes the loop too flexibile to get the original stuctures by NMR or X-ray. Moreover, for β2, it is impossible to get the correctly folded β2 without associating α subunit as β2 cannot traffic to the membrane surface alone. An α subunit may associate with a variable number of β2 subunits (up to four β2 subunits), such that a symmetrical structure of the complex as one channel cannot be ensured for crystallization. These intrinsic factors present considerable challenges to fully resolve the crystal structure of a transmembrane α/β2 complex and to validate the correct structure of β2 without information about the pairing mode of its cysteines.

Previous studies revealed that the extracellular loop of the β auxiliary subunits determined toxin binding and was located near the pore [17]; moreover, the degree of outward rectification of the BK channel is dominated by the positively charged rings of the β2 extracellular loop located at the outer entrance of the channel pore [26]. Based on the changes in rectification ratios, association, and charge distribution caused by the site-directed single and double cysteine mutations, we revealed that the association of mSlo1/β2 was extremely sensitive to the disulfide-bond disruption of four conserved cysteines, indicating that there were two clearly conserved pairs, C1(C84)-C5(C113) and C6(C142)-C8C(174), and a non-conserved pair, C3(C101)-C7(C148), among the eight cysteines. However, the last pair of C2(C97) and C4(C105) still remains uncertain, although molecular modeling indicated that these two cysteines were in proximity. The potential pairing might be regulated by the level of glycosylation at the site between C2 and C4.

Thus, we were able to successfully validate three cysteine pairings, as identified by electrophysiological analysis of mutants. Because TEV is located between C5 and C6, the whole loop could not be cut into two segments by the TEV protease, except for the C3S or the C7S mutant (Figures 7 and S6); this finding indicates that C3 pairs with C7, and neither C6 nor C8 can pair with C1, C2, C4 or C5, consistent with our electrophysiological results showing that the cysteines pair together within a subgroup *i.e*., C1(C84)-C5(C113) and C6(C142)-C8C(174).

In summary, we developed a novel approach combining site-directed mutagenesis, electrophysiology and computation to probe the structural information of BK channels. the rectification of BK(β2) currents and TEV protease digestion were chosen to determine the pairing mode of cysteines. We identified at least three pairs of cysteines in the β2 extracellular loop; these pairs may be important for stabilizing the extracellular domain of the β2 loop and may impart BK channels with distinct functionality. Although this model needs future validation of X-ray crystal or NMR structure of the β2 loop, the Cys-Cys pairing mode in the β2 loop in this model might be informative and potentially applicable to studies of BK and other ion channels. Our structural model may also help us resolve the structural conformation of β2 and potentially develop new drugs that target extracellular domain of specific BK channels in the future.

## Materials and methods

### Constructs and mutations

The BK α constructs were generated from the mbr5 splice variant of mouse *Slo1* (*KCNMA1*, GenBank accession number L16912). The cDNA of Human *β1* (KCNMB1, GenBank accession number U25138) and human *β2* (*KCNMB2*, GenBank accession number AF209747.1) were subcloned into the pcDNA3.1(+) vector. For Western blot experiments, an epitope HA tag (hemagglutinin, YPYDVPDYA) was added at the C-termini of β1 (*i.e*., C-HA tagged β1) and β2 (*i.e*., C-HA tagged β2). For protease digestion experiments, a TEV (*tobacco etch virus*) protease recognition site (ENLYFQG) was inserted after the 137^th^ amino acid of β2 (137TEV β2), this residue could be precisely recognized and was used to quantify the surface expression of β2 subunits [27]. The C-HA-tagged 137TEV β2 acts as a pseudo-WT (pWT) β1 and β2 in protease digestion experiments. Mutations were introduced using a QuickChange Site-Directed Mutagenesis Kit (Stratagene). All constructs and point mutations were verified by direct DNA sequence analysis.

### Western blot

The C-HA-tagged human β1 or β2 and their mutants in pcDNA3.1 were expressed in HEK293 cells. 24 hours after transfection, the cells were lysed using the lysis buffer (20 mM Tris-HCl, pH 7.5, 150 mM NaCl, 1% NP-40, 0.5% Triton X-100, 0.2 mM phenylmethylsulfonyl fluoride and protease inhibitors). For protease digestion, 10U TEV protease was added into the lysis buffer. After vertical rotation at 4°C for 12 h, the lysed cells were centrifuged at 12,000 rpm at 4°C for 30 min. Proteins in the lysate were then divided into two equal parts, and treated with 5× loading buffer containing ± DTT (DL-dithiothreitol), which was used to reduce the disulfide bonds in proteins. The treated samples were then incubated at 50 °C for 10 min, centrifuged at 12,000 rpm at room temperature for 10 min, separated on polyacrylamide gels, and transferred to a nitrocellulose membrane. After being blocked with 5% nonfat milk in 0.1% Tween 20 in Tris-buffered saline, these blots were probed with primary antibody directed against the HA tag. Horseradish peroxidase-coupled mouse anti-rabbit IgG was used as the secondary antibody for β2 blots. The membrane was washed with 0.1% Tween 20 in Tris-buffered saline, and proteins were visualized using an enhanced chemiluminescence detection system.

### Cell culture and transient transfections in HEK293 cells

HEK293 cells were cultured in DMEM supplemented with 10% fetal bovine serum (FBS), 100 U/ml penicillin and streptomycin in incubators at 37°C and 5% CO_2_. One day before transfection, the cells were transferred to 24-well plates. At 90% confluence, transfections were performed using Lipofectamine 2000 (Invitrogen). Four to six hours after transfection, the cells were transferred to a slide coated with poly-D-lysine (Sigma) for the patch-clamp recordings. For all the co-transfections, the plasmid mass ratio of α to β subunits was 1:2.

### Patch clamp recording

For recordings, transfected HEK293 cells were transferred one day after transfection to a bath solution containing 160 mM MeSO_3_K, 10 mM HEPES, 5 mM N-hydroxyethylenediaminetriacetic acid (HEDTA) with added Ca^2+^ to achieve 10 mM free Ca^2+^, defined by the EGTAETC program (McCleskey, Vollum Institute, Portland), and pH 7.0. All experiments were performed at room temperature using excised patches in the inside-out recording configuration. The pipette solution consisted of 160 mM K^+^ solution containing 160 mM MeSO_3_K, 2 mM MgCl_2_, and 10 mM HEPES (pH 7.0). Patch pipettes were pulled from borosilicate glass capillaries with resistances of 2–3 megaohms when filled with pipette solution. The experiments were performed using an AXON-200B patch-clamp amplifier and its accompanying software (Axon Instruments, Inc., USA). Currents were typically digitized at 20 kHz and filtered at 8.5 kHz.

### Mathematical analysis of the rectification ratio

The recording data were analyzed using softwares Clampfit (Axon Instruments, Inc.) and Sigmaplot (SPSS, Inc.). Unless otherwise stated, data were presented as the mean ± S.D. V_50_ was defined as the voltage at which the conductance was half of the maximum conductance by fitting the G-V curve using the Boltzmann equation: G/G_max_ = (1 + exp((V − V_50_)/κ))^−1^, where κ is a factor affecting the steepness of the activation. The gray level of the protein sample strip on the blot picture was analyzed using ImageJ software (National Institutes of Health, USA).

The rectification ratio (*R*) was defined as *R* = |I_100_/I_−100_|, where I_100_ or I_−100_ represented the currents activated at either +100 mV or −100 mV. The rectification ratio of individual BK(mSlo1) (*i.e., R*_α_) was previously determined to be equal to 1.2, and that of BK(mSlo1+ β2) (*i.e., R*_β2_) was experimentally determined to be equal to 1.8 in the case of a β2 binding to α [26], respectively (Figure 1). Thus, the ratio *R* was suggested to be almost proportional to the molecular number of β2 binding to mSlo1 α channel. Here, we define that the binding probability of β2 to mSlo1 α is p, the unbinding probability of β2 is q, which is equal to 1-p. When one cysteine of the β2 extracellular loop is mutated (hereinafter, β2* was denoted as a β2 mutant), the p values will be significantly reduced, causing incomplete inactivation that led to a large “offset”, as shown in Figure 3. Thus, the measured rectification ratio of the mutant (*R*_β2*_) is smaller because some BK channels do not associate with or just associate with β2 to a smaller extent. To better compare *R*_β2*_ with *R*_β2_, we introduce an equivalent ratio, *i.e*., corrected to four β2 per BK, *R*_eq_ = *R*_α_+4Δx, where the Δx is a linear increase of *R* per β2. Therefore, we can probe the changes in *R* or associations of α to β (or the p values) among different β2 mutants (β2*). Assuming that the combination of β2 binding to α obeys to a binomial distribution, Equation (1) is obtained as follows:

(1) $$R_{\alpha }C_{4}^{0}p^{0}q^{4}+\left(R_{\alpha }+\Delta x\right)C_{4}^{1}p^{1}q^{3}+\left(R_{\alpha }+2\Delta x\right)C_{4}^{2}p^{2}q^{2}+\left(R_{\alpha }+3\Delta x\right)C_{4}^{3}p^{3}q^{1}+\left(R_{\alpha }+4\Delta x\right)C_{4}^{4}p^{4}q^{0}=R_{\beta 2*}$$

where $C_{4}^{n}$ (n = 0, 1, 2, 3, 4) are basic coefficients of binomial distribution.

Simplifying Equations (1), (2) and (3) are got as listed below:

(2) $$\Delta x=\frac{R_{\beta 2*}-R_{\alpha }}{4p}$$

(3) $$R_{eq}=R_{\alpha }+\frac{R_{\beta 2*}-R_{\alpha }}{p}$$

Specifically, for a completed inactivation (p = 1), *R*_eq_ is equal to *R*_β2*_. In Figure 3(a), we displayed a complete inactivation current of the BK(mSlo1+ β2) complex channel, indicating that BK channel associated with four β2 subunits to yield *R*_β2_ equal to 1.8. In Figure 3(b), we demonstrated an incomplete inactivation current derived from one cysteine mutation (C1S, also called as C84S) in the β2-loop region. This current trace showed an offset C. To calculate the q value of this complex channel, the entire trace is fitted using Equation (4), including the activation and inactivation, to a double exponential function f(t) as follows:

(4) $$f\left(t\right)=A_{1}*exp\left(-\frac{t}{{\;}_{1}}\right)+A_{2}*exp\left(-\frac{t}{{\;}_{2}}\right)+C$$

where A_1_ is denoted as the amplitude of the inactivation term, and τ_1_ is the inactivation time constant. A_2_ is denoted as the amplitude of the activation term, τ_2_ is the activation time constant, and C is the steady-state term (or non-inactivating pedestal current). The fitting parameters of mSlo1+ β2 (C1S) are shown in the bottom of Figure 1(b), which will generate Equation (5):

(5) $$q={\left(\frac{C}{A}\right)}^{\frac{1}{4}}$$

where A = max(abs(A_1_), abs(A_2_)), which is consistent with I_max_ in the HH model [7].

### Structure prediction of the β2 loop and assembly of the BK-β2 complex

After the information of disulfide bonds was available, three-dimensional structure of the extracellular β2 loop could be predicted by Rosetta (version 2.3.1) [34] suites with disulfide bonds constraints. 10k decoys were produced during the *ab initio* protein folding task based on the sequence information. To obtain coarse decoys, the structural sampling was first performed in low-resolution mode. Then, the resulting models were clustered into 280 different clusters around the corresponding centers using TOP_CLUSTER_SIZE 199, and the cluster centers were relaxed according to the full-atom structure refinement. The most preferred models were used for further construction of the full-length β2 subunit.

The structural model of the full–length β2 subunit was constructed by connecting the predicted extracellular β2 loop, the two transmembrane helices and the N-terminus (PDB code 1JO6) [35]. The tetrameric hSlo1 channel was built using the latest cryo-EM structures of the high conductance Ca^2+^ activated K^+^ channel (PDB codes 5TJ6 and 5TJI) [36,37] as multi-homology templates with highly overall sequence similarity, using the common homology modeling protocol. BKα subunits interact with β2 subunt at mole ratio of 4:1, the complex of BKα and β2 was then assembled manually and the only one β2 subunit was used to clearly show the orientation and special positive charged residues. The S0, S1, S2 helix of BKα and the TM1 and TM2 of β2 was orientated according to the previous reports [38,39]. Due to their flexibility, the N-terminal loop (with a sequence of MDALIIPVTMHEVPCDSRGQR) and the long loops, with the residue sequences of PKRIKKCGCKRPKMSIYKRMRRACCFD

CGRSERDCSCMSGRVRGNVDTLERAFPLSSVSVNDCSTSFRAFEDEQPSTLSPKKKQRNGGMRNSPNTSPKLMRHDPLLIPGNDQIDNMDSNVKKYDST and HC

GGKTKEAQKINNGSSQADGTLKPVDEKEEAVAAE, were deleted during construction of both structural models. The final complex model of BKα/β2 was refine by Amber14 with ff14SB force field in generalized born implicit Solvent(GBIS). After 20,000 steps minimization for the coarse complexes, followed by 1,000,000 heating steps from 0K to 325 K, the ensembles were finally under 50,000,000 steps equilibrations.Final stable complex models were extracted from the trajectories of production simulations and analyzed in detail as general [34].The final model was extracted from the simulation trajectory.
