# Supplementary material for: Rectification ratio based determination of disulfide bonds of β2 extracellular loop of BK channel
Source: Channels (Austin). 2018 Nov 26;13(1):17–32. doi: 10.1080/19336950.2018.1551660 (PMC6298698; doi:10.1080/19336950.2018.1551660)
Supplement: Supplemental Material [file kchl-13-01-1551660-s001.doc]

**Supplementary file**

**Putative disulfide crosslinking pattern of the extracellular loop of the β2 subunit of BK channel**

Xiyimg Guo^1¶^, Haowen Liu^1¶^, Zhigang Huang^1^, Yanting Wang^1,2^, Yan Zhang^1^, Lu-Yang Wang^3^, Chunyang Cao^2^,Sheng Wang^1^, Jiuping Ding^1^

^1^Key Laboratory of Molecular Biophysics of the Ministry of Education, College of Life Science and Technology, Huazhong University of Science and Technology, Wuhan, Hubei, China

^2^State Key Laboratory of Bio-organic and Natural Product Chemistry, Shanghai Institute of Organic Chemistry, Chinese Academy of Sciences, 345 Lingling Road, Shanghai, 200032, China

^3^Program in Neurosciences and Mental Health, SickKids Research Institute and Department of Physiology, University of Toronto, Toronto, Canada

Address correspondence to:

Sheng Wang,

Tel. 86-27-87793053; Fax: 86-27-87792337, E-mail: shengwang@hust.edu.cn.

Jiuping Ding,

Tel. 86-27-87792153; Fax: 86-27-87792337, E-mail: jpding@mail.hust.edu.cn.

^¶^These authors contributed equally to this work

**Table S1** **The kinetic influence of the loop mutations on the BK(β2)**

| BK(β2*) | V_50_(mV) | τ_i(100mV)_(ms) | R_eq_ |
| --- | --- | --- | --- |
| mSlo1/β2 | -12.7±4.8 | 17.0±0.5 | 1.84±0.03 |
| mSlo1/β2(C1S) | 5.2±3.2 | 26.1±1.2 | 1.38±0.03 |
| mSlo1/ β2(C2S) | -13.7±6.2 | 15.2±0.8 | 1.44±0.04 |
| mSlo1/β2(C3S) | -11.5±5.5 | 16.4±0.5 | 1.35±0.01 |
| mSlo1/β2(C4S) | -10.5±4.5 | 16.3±0.5 | 1.44±0.02 |
| mSlo1/β2(C5S) | 11.5±6.5 | 28.5±0.9 | 1.35±0.05 |
| mSlo1/β2(C6S) | 10.2±6.2 | 24.6±0.7 | 1.38±0.05 |
| mSlo1/β2(C7S) | -12.7±4.8 | 18.0±0. 5 | 1.41±0.01 |
| mSlo1/β2(C8S) | 14.1±4.9 | 25.0±0.6 | 1.37±0.05 |
| mSlo1/β2(C1S,C2S) | 12.0±6.1 | 27.2±0.7 | 1.39±0.03 |
| mSlo1/β2(C1S,C3S) | 12.4±5.8 | 28.1±0.9 | 1.41±0.03 |
| mSlo1/β2(C1S,C4S) | 11.3±6.3 | 29.2±1.0 | 1.38±0.02 |
| mSlo1/β2(C1S,C5S) | 18.4±6.1 | 30.2±1.4 | 1.36±0.04 |
| mSlo1/β2(C1S,C6S) | 14.3±5.1 | 28.4±0. 8 | 1.42±0.05 |
| mSlo1/β2(C1S,C7S) | 13.5±6.2 | 27.5±0.9 | 1.38±0.04 |
| mSlo1/β2(C1S,C8S) | 14.0±6.1 | 27.2±1.0 | 1.42±0.04 |
| mSlo1/β2(C2S,C3S) | -10.7±4.2 | 18.0±0.7 | 1.36±0.04 |
| mSlo1/β2(C2S,C4S) | -12.5±4.8 | 16.9±0.6 | 1.26±0.04 |
| mSlo1/β2(C2S,C5S) | 13.4±5.6 | 26.2±0.9 | 1.37±0.04 |
| mSlo1/β2(C2S,C6S) | 12.7±5.4 | 27.5±0.7 | 1.37±0.05 |
| mSlo1/β2(C2S,C7S) | -9.7±4.5 | 18.2±0.5 | 1.60±0.03 |
| mSlo1/β2(C2S,C8S) | 12.1±5.8 | 29.3±1.0 | 1.38±0.04 |
| mSlo1/β2(C3S,C4S) | -10.2±4.8 | 17.1±0.4 | 1.46±0.02 |
| mSlo1/β2(C3S,C5S) | 12.6±5.3 | 29.2±0.9 | 1.38±0.03 |
| mSlo1/β2(C3S,C6S) | 11.7±6.1 | 26.4±0.7 | 1.35±0.03 |
| mSlo1/β2(C3S,C7S) | -8.9±4.5 | 19.1±0.5 | 1.34±0.04 |
| mSlo1/β2(C3S,C8S) | 14.5±5.6 | 25.8±0. 7 | 1.40±0.04 |
| mSlo1/β2(C4S,C5S) | 10.7±4.8 | 28.4±0.8 | 1.35±0.04 |
| mSlo1/β2(C4S,C6S) | 12.4±5.7 | 26.7±0.8 | 1.39±0.04 |
| mSlo1/β2(C4S,C7S) | -11.2±4.8 | 17.5±0.5 | 1.33±0.05 |
| mSlo1/β2(C4S,C8S) | 12.5±6.2 | 27.5±0.8 | 1.38±0.04 |
| mSlo1/β2(C5S,C6S) | 13.7±5.8 | 30.2±1.3 | 1.41±0.04 |
| mSlo1/β2(C5S,C7S) | 12.7±5.1 | 27.4±0.7 | 1.36±0.04 |
| mSlo1/β2(C5S,C8S) | 13.1±5.3 | 28.2±0.9 | 1.42±0.02 |
| mSlo1/β2(C6S,C7S) | 9.7±5.2 | 25.2±0.7 | 1.40±0.05 |
| mSlo1/β2(C6S,C8S) | 14.4±5.6 | 26.7±0. 8 | 1.38±0.05 |
| mSlo1/β2(C7S,C8S) | 12.0±4.8 | 27.3±0.9 | 1.37±0.04 |
| mSlo1/β2(Q83K) | -12.0±4.5 | 18.7±0.5 | 1.88±0.03 |
| mSlo1/β2(N96K) | -9.8±4.8 | 20.2±0.5 | 2.45±0.08 |
| mSlo1/β2(S100K) | -12.4±4.2 | 18.1±0.4 | 2.13±0.03 |
| mSlo1/β2(S100D) | -11.2±4.4 | 19.2±0.5 | 1.68±0.04 |
| mSlo1/β2(S100K,K147A) | -10.7±4.1 | 17.5±0.5 | 1.69±0.04 |
| mSlo1/β2(S100K,K147D) | -11.9±4.6 | 19.5±0.7 | 1.85±0.05 |
| mSlo1/β2(D104A) | -10.5±4.6 | 17.0±0.7 | 1.71±0.07 |
| mSlo1/β2(L114K) | 5.7±5.4 | 24.2±0.8 | 1.85±0.05 |
| mSlo1/β2(S173K) | -12.2±4.2 | 18.5±0.5 | 2.13±0.02 |
| mSlo1/β2(S173D) | -11.3±4.6 | 17.6±0.4 | 1.69±0.02 |
| mSlo1/β2(S173K,K141A) | -12.0±4.3 | 18.2±0.6 | 1.88±0.03 |
| mSlo1/β2(S173K,K141D) | -12.8±4.5 | 17.5±0.6 | 1.65±0.05 |

**Table S2 R_eq_ values for all combinations of C→S mutations**

|  | **C1S** | **C2S** | **C3S** | **C4S** | **C5S** | **C6S** | **C7S** | **C8S** |
| --- | --- | --- | --- | --- | --- | --- | --- | --- |
| **C1S** | 1.38±0.03 |  |  |  |  |  |  |  |
| **C2S** | 1.39±0.03 | 1.44±0.04 |  |  |  |  |  |  |
| **C3S** | 1.40±0.03 | 1.37±0.04 | 1.35±0.01 |  |  |  |  |  |
| **C4S** | 1.38±0.02 | 1.26±0.04 | 1.46±0.02 | 1.44±0.02 |  |  |  |  |
| **C5S** | 1.36±0.04 | 1.37±0.04 | 1.38±0.03 | 1.35±0.04 | 1.35±0.05 |  |  |  |
| **C6S** | 1.42±0.05 | 1.37±0.05 | 1.35±0.03 | 1.39±0.04 | 1.41±0.04 | 1.38±0.03 |  |  |
| **C7S** | 1.38±0.04 | 1.61±0.03 | 1.34±0.04 | 1.33±0.05 | 1.36±0.04 | 1.40±0.05 | 1.41±0.01 |  |
| **C8S** | 1.42±0.04 | 1.38±0.04 | 1.40±0.04 | 1.38±0.04 | 1.42±0.02 | 1.38±0.05 | 1.37±0.04 | 1.37±0.05 |

**Figure S1**

**
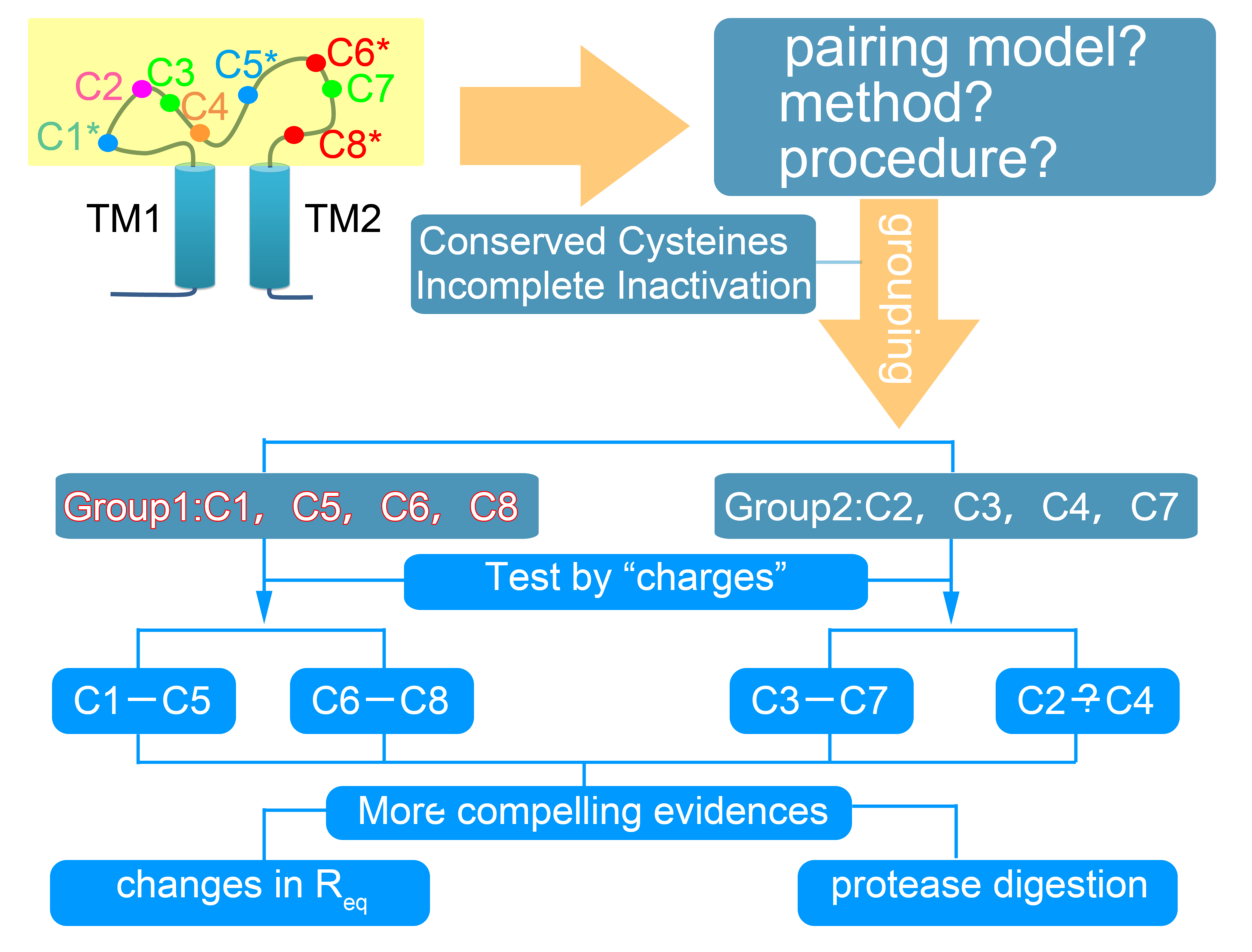
**

**Figure S1 A schematic diagram drawing for seeking out the possible cys-cys pairs among eight cysteines in the β2 extracellular loop.** The extracellular loop of β2 contains both of the conservative (C1, C5, C6 and C8, labeled an asterisk) and non-conservative (C2, C3, C4 and C7 in cyan) cysteines, of which the conservative ones resulted in an incomplete inactivation based on the Cys→Ser (or C→S) mutation, differing from that of the non-conservative ones. With the help of the changes in rectification (Req) characteristics, induced by changing the charges surrounding cysteins or directly by the C→S mutations, and the protease digestion to finally determine their pairing modes.

**Figure S2**


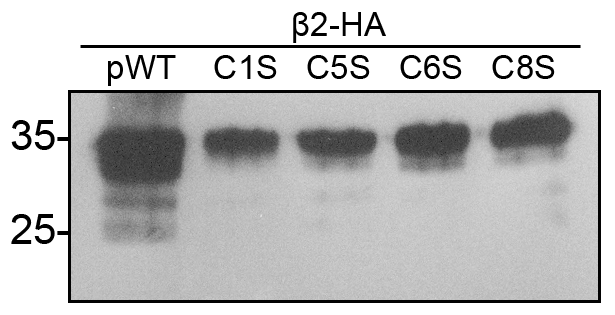


**Figure S2 The membrane expression conditions of β2-HA and its related mutants.** All the constructs were expressed in HEK293 cells; 24 h after transfection, the membrane fractions were extracted by Membrane Protein Extraction Kit (Sangon Biotech (Shanghai, China) Co., Ltd). Proteins in the membrane fractions were separated on polyacrylamide gels, and transferred to a nitrocellulose membrane. All lanes showed the same major band of about 35 kDa. Part of the lanes showed additional bands about 32 kDa, 28 kDa, 25 kDa. These additional bands represent different levels of glycosylation of β2. The mutants β2-C1S-HA, β2-C5S-HA, β2-C6S-HA, β2-C8S-HA could be targeted in membrane, although they showed incomplete inactivation when they co-expressed with mSlo1, respectively.

**Figure S3**


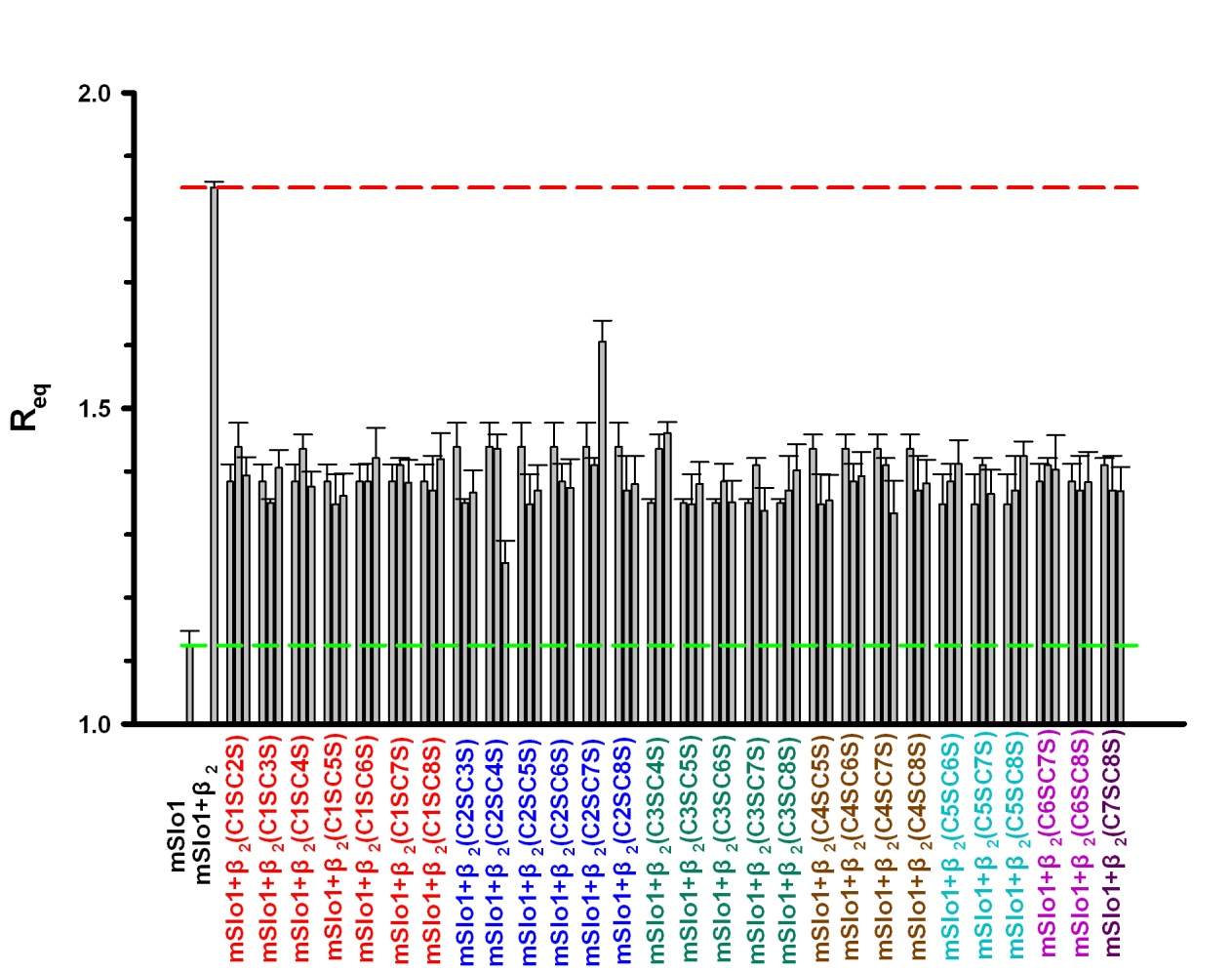


**Figure S3 Comparison of** **the R_eq_ values for all combinations of C→S mutations.** Except for two single bars for mSlo1 and mSlo1+β2, as indicated, each group was composed of three bars and is labeled as mSlo1+β2(CiSCjS) (i, j=1, 2,…8); these groups represent three classes of mutants, β2(CiS), β2(CjS) and β2(CiSCjS), shown from left to right, respectively. The statistical significance for each group was determined using one-way ANOVA, and the results were not significant (p>0.05) except for the mSlo1+β2(C2SC4S) and mSlo1+β2(C2SC7S) results. All the R_eq_ values for all the combinations of C→S mutations are shown in Table S2.

**Figure S4**


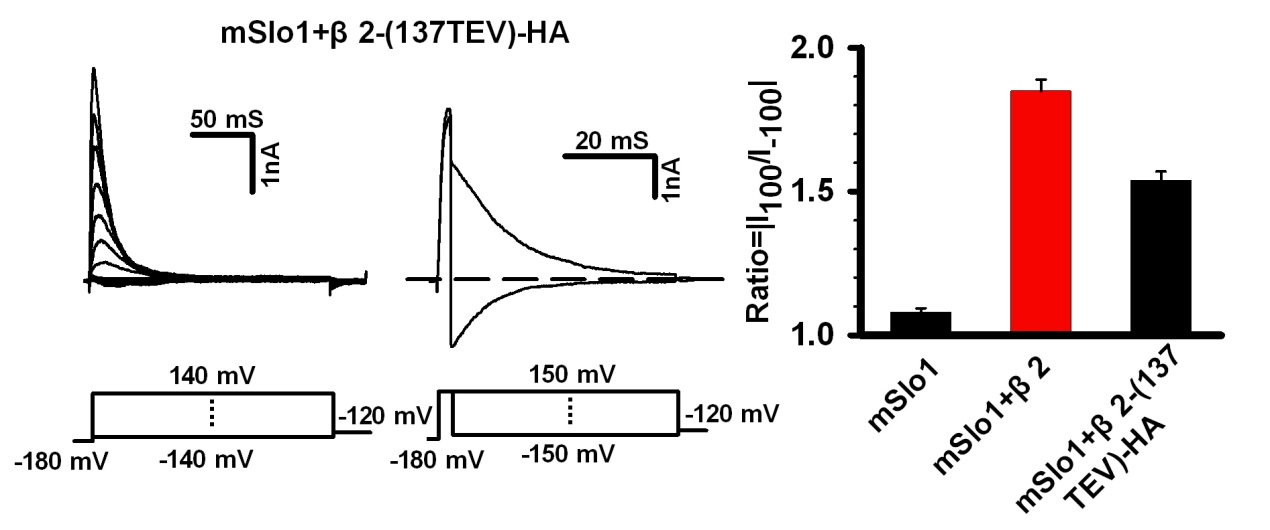


**Figure S4 Rectification characteristics of mSlo1+β2-(137TEV)-HA channels.** Left, representative traces of mSlo1+β2-(137TEV)-HA channels, recorded from inside-out patches from HEK293 cells. The protocol is plotted at the bottom. Middle, instantaneous tail currents were obtained from the same patch as shown in the left panel. The protocol is plotted at the bottom, and only ±100 mV stimulations are shown. Right, the histogram of rectification ratios R=|I_100_/I_-100_| is plotted for mSlo1+β2 and mSlo1+β2-(137TEV)-HA channels. The ratios are 1.85±0.03 (n=12) and 1.54±0.04 (n=6) for mSlo1+β2 and mSlo1+β2-(137TEV)-HA, respectively.

**Figure S5**

**
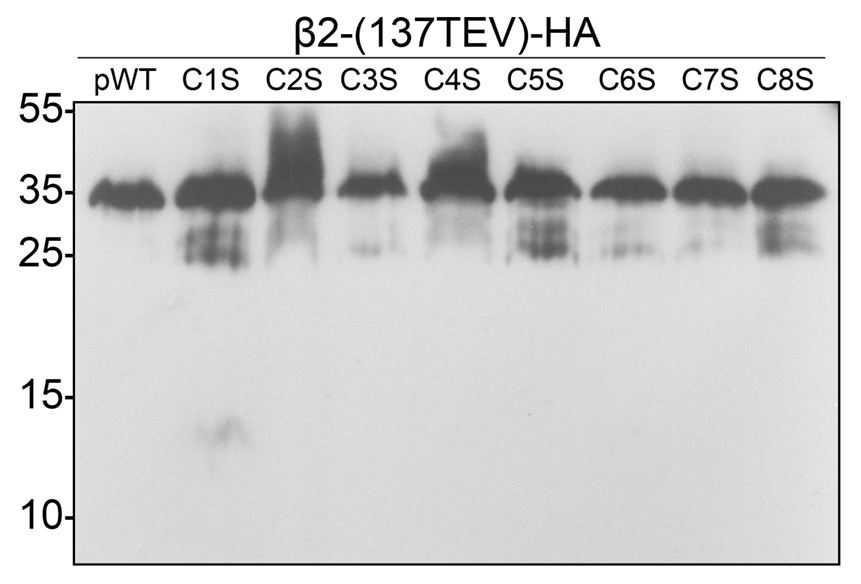
**

**Figure S5 Immunoblot (IB) with anti-HA antibody showing DTT treated** **β2-(137TEV)-HA and all types of single cysteine mutants.** All constructs were expressed in HEK293 cells; 24 h after transfection, the cells were lysed (lysis buffer do not contain TEV protease). Proteins in the lysate treated with 5× loading buffer containing DTT. The treated samples were then incubated at 50 °C for 10 min, centrifuged at 12,000 rpm at room temperature for 10 min, separated on polyacrylamide gels, and transferred to a nitrocellulose membrane. All lanes showed the same major band of about 35 kDa. Part of the lanes showed additional bands about 32 kDa, 28 kDa, 25 kDa. These additional bands represent different levels of glycosylation of β2.

**Figure S6**

**
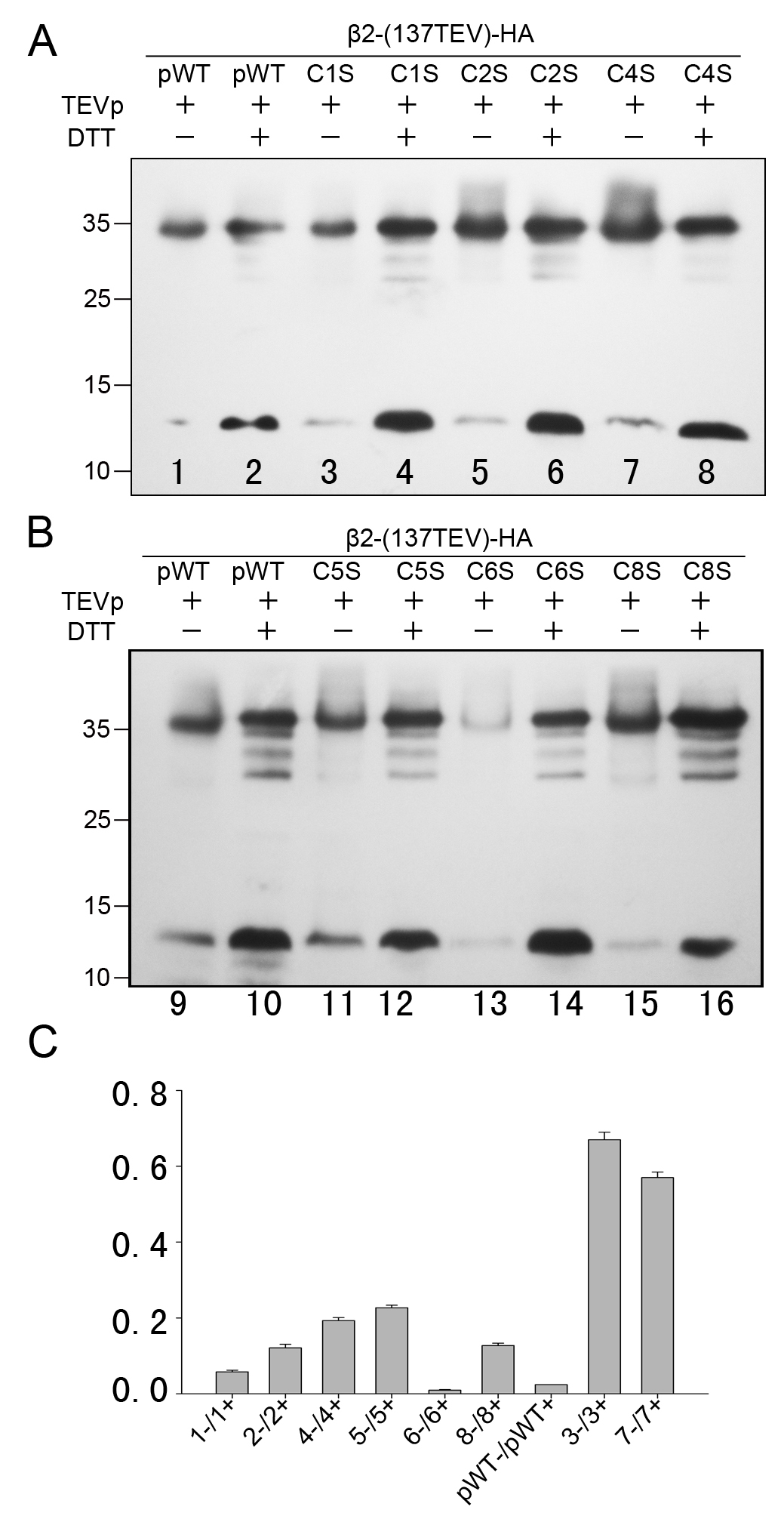
**

**Figure S6 Additional evidence for cysteine pairs derived from the restriction enzyme digestion experiments at 137TEV.** For mutants C1S, C2S, C4S, C5S, C6S, C8S, when after both TEV protease and DTT treated, they could show an additional band about 13 kDa. The relative gray levels of the 13 kDa bands between the two lanes of ± DTT are plotted as indicated. Their ratios are 0.057±0.005 (n=3) for C1S(-DTT)/C1S(+DTT), 0.121±0.010 (n=3) for C2S(-DTT)/C2S(+DTT), 0.193±0.008 (n=3) for C4S(-DTT)/C4S(+DTT), 0.227±0.007 (n=3) for C5S(-DTT)/C5S(+DTT), 0.009±0.002 (n=3) for C6S(-DTT)/C6S(+DTT), 0.127±0.006 (n=3) for C8S(-DTT)/C8S(+DTT), 0.024±9.4e-6 (n=3) for pWT(-DTT)/pWT(+DTT), 0.67±0.020 (n=3) for C3S(-DTT)/C3S(+DTT) and 0.57±0.015 (n=3) for C7S(-DTT)/C7S(+DTT). Data are shown as the mean ± s.e.m. Statistical significance for the data was determined with Student’s t-test (**P <0.01).

**Figure S7**


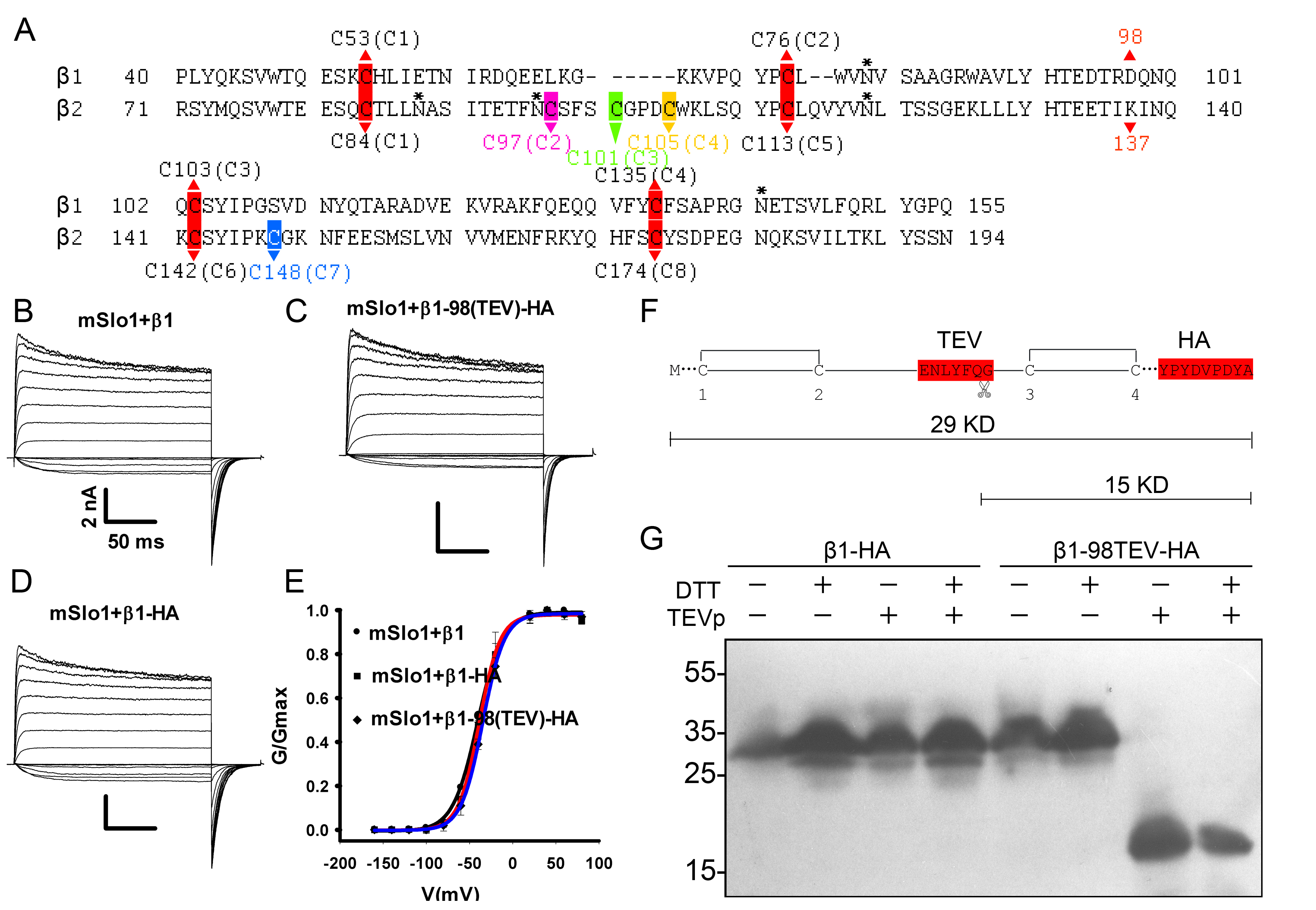


**Figure S7 Confirmation of disulfide cross-linking pattern of cysteines in the hβ1 extracellular loop.** **(A)** Conservation analysis of extracellular loops of β1 and β2, the glycosylation sits are marked with *, the conserved cysteine amino acid residues are marked with red background. (**B**) Representative traces of mSlo1+β1. (**C**) Representative traces of mSlo1+β1-98(TEV)-HA. (**D**) Representative traces of mSlo1+β1-HA. All voltage protocals were the same as Fig.2C. (**E**) G-V curves were plotted for mSlo1+β1 (●), mSlo1+β1-98(TEV)-HA (♦) and mSlo1+β1-HA (■). The V_50_s were -45.1±5.4 mV, 40.7±6.3 mV and 44.5±5.0 mV for mSlo1+β1,mSlo1+β1-98(TEV)-HA and mSlo1+β1-HA, respectively. Solid lines are single Boltzmann functions fitted to each G-V curve. (**F**) Predicted cysteine-pairing model of the pWT(β1) tagged with 137TEV and C-HA. The relative positions of the cysteine residues in the hβ1 extracellular loop, the TEV protease recognition site, and the HA tag are labeled as indicated. In this model, the molecular weight of pWT β1 is approximately 29kDa, including two N-linked glycosylation sites, and the TEV protease proteolytic fragment with the remaining C-terminal HA epitope is approximately 15kDa, including a N-linked glycosylation site. (**G**) Immunoblot (IB) with anti-HA antibody showing the fragments containing the C-terminal HA epitope of β1. Top, an approach was designed for TEV enzyme restriction analysis.
